# Multifunctional Core-Shell NiFe_2_O_4_ Shield with TiO_2_/rGO Nanostructures for Biomedical and Environmental Applications

**DOI:** 10.1155/2022/4805490

**Published:** 2022-05-30

**Authors:** R. Esther Nimshi, J. Judith Vijaya, B. Al-Najar, L. Hazeem, M. Bououdina, L. John Kennedy, K. Kombaiah, S. Bellucci

**Affiliations:** ^1^CNR Laboratory, Department of Chemistry, Loyola College, University of Madras, Chennai 34, India; ^2^Department of Physics, College of Science, University of Bahrain, P.O. Box 32038, Zallaq, Bahrain; ^3^Department of Biology, College of Science, University of Bahrain, P.O. Box 32038, Zallaq, Bahrain; ^4^Department of Mathematics and Science, Faculty of Humanities and Sciences, Prince Sultan University, Riyadh, Saudi Arabia; ^5^Materials Division, School of Advanced Sciences, Vellore Institute of Technology University, Chennai Campus, Chennai 127, India; ^6^Department of Chemistry, Arul Anandar College, Kamaraj University, 625 514 Madurai, India; ^7^INFN-Laboratori Nazionali di Frascati, Via E. Fermi 54, 00044 Frascati, Italy

## Abstract

Multifunctional core@shell nanoparticles have been synthesized in this paper through 3 stages: NiFe_2_O_4_ nanoparticles by microwave irradiation using *Pedalium murex* leaf extract as a fuel, core@shell NiFe_2_O_4_@TiO_2_ nanoparticles by sol-gel, and NiFe_2_O_4_@TiO_2_@rGO by sol-gel using preprepared reduced graphene oxide obtained by modified Hummer's method. XRD analysis confirmed the presence of both cubic NiFe_2_O_4_ spinel and tetragonal TiO_2_ rutile phases, while Raman spectroscopy analysis displays both *D* and *G* bands (*I*_*D*_/*I*_*G*_ = 1.04) associated with rGO. Morphological observations by HRTEM reveal a core-shell nanostructure formed by NiFe_2_O_4_ core as confirmed by SAED with subsequent thin layers of TiO_2_ and rGO. Magnetic measurements show a ferromagnetic behavior, where the saturation magnetization drops drastically from 45 emu/g for NiFe_2_O_4_ to 15 emu/g after TiO_2_ and rGO nonmagnetic bilayers coating. The as-fabricated multifunctional core@shell nanostructures demonstrate tunable self-heating characteristics: rise of temperature and specific absorption rate in the range of Δ*T* = 3–10°C and SAR = 3–58 W/g, respectively. This effectiveness is much close to the threshold temperature of hyperthermia (45°C), and the zones of inhibition show the better effective antibacterial activity of NTG against various Gram-positive and Gram-negative bacterial strains besides simultaneous good efficient, stable, and removable sonophotocatalyst toward the TC degradation.

## 1. Introduction

In the modern research world, multifunctional nanoparticles (NPs) have attracted much attention with numerous fascinating properties and potential diverse applications in optics, magnetic, electronics, and catalysis. Single functional NPs must overcome the requirements for numerous applications such as water purification [1], biosensing [2], and cancer treatment [3, 4]. In this context, the proposed solution consists of a combination of two or more different functional nanomaterials to form multifunctional nanoparticles [5, 6]. Nowadays, the whole world is facing human health issues and environmental pollution. Generally, we are focusing on human health issues more than environmental problems because pharmaceutical companies synthesize more and more medicinal products; this medicinal side products and wastes are also one of the causes of environmental pollution. Hence, the product should have human therapeutic nature as well as environmental pollution-treated nature. It is very essential to this world. Specifically, core@shell NPs have attracted great attention due to the distinctive properties originating from a well-engineered and good functional choice of materials; thereby, the fabricated nanostructures demonstrate promising novel applications [7, 8].

Hyperthermia treatment has been identified as one of the potential cancer treatments, which depends on raising the temperature of the tumor cells up to around 42–47°C, which is enough to destroy the cancer cells if applied for at least 10 minutes [9]. Magnetic NPs are good functional materials for hyperthermia cancer treatment due to their biocompatibility and nontoxicity and can be remotely heated by applying an external magnetic field [10]. This heating ability has also been used in different biomedical applications, such as magnetic separation and target drug delivery [11, 12]. The induction heating ability of NPs is usually estimated by the specific absorption rate (SAR) within the medium.

Normally, the huge global antibiotic usage per year is near 200,000 tons of medical compounds. Tetracycline is one of the most required antibiotics. The discharge of antibiotics from hospitals' effluents, municipal wastewaters, and drugs' industrial drainage have been identified as very polluting to the natural environment and ecosystems. Antibiotics possess a low biodegradability, high toxicity, and high-water solubility in nature and hence can be easily absorbed by humans as well as animals [13]. Even at low concentrations of antibiotics, their discharge into the environment leads to teratogenic problems in pregnant women, function disorders of endocrine glands, and chronic toxicity [14, 15]. Hence, it can produce antibiotic resistance genes and bacteria. A variety of methods have been adopted for the antibiotics' removal from water bodies. In the photocatalytic process, renewable sources such as solar energy utilize visible light photocatalysis, and ultrasonic irradiation is the most important and efficient method. However, the sonophotocatalytic approach by coupling the two processes boosted the antibiotics' degradation [16–18].

The magnetic energy of magnetic NPs, which is transformed into thermal energy, shows promising application behavior in hyperthermia treatment [19]. Magnetic transition metal ferrites with a general formula MFe_2_O_4_ (M = Ni, Co, etc.) can be written based on ions distribution A^3+^[B^2+^, B^3+^]O_4_^2−^, where tetrahedral cation sites are denoted by “A” and octahedral cation sites are denoted by “B.” The Fe^3+^ cations occupy half of the tetrahedral A-sites, and the rest of the Fe^3+^ cations and divalent M^2+^ cations occupy the octahedral site in the spinel structure, resulting in improved conductivity. Among the different metal ferrites, nickel ferrite (NiFe_2_O_4_) exhibits soft magnetic semiconducting properties with ferromagnetic behavior, better chemical stability, low conductivity, and catalytic performance [20, 21]. They are used in many fields such as catalyst carrier, rectangular hysteresis material, microwave absorbing, and supercapacitor. Magnetic NPs are aggregating in nature because of their high surface energy and strong van der Waals interactions. The formation of a shell belonging to another functional material on a spinel ferrite surface has been proposed as an alternative approach for better control of NPs aggregation and novel functional abilities toward a broad range of applications [22].

TiO_2_ is one of the remarkable functional materials used in photocatalysis, photovoltaic cells, photodegradation, electrochromic devices, white pigment in paint, cosmetics, and food coloring due to its strong optical absorption, high chemical stability, nontoxicity, and low cost [23–25]. Among all applications, TiO_2_ has been undoubtedly recognized as a promising functional material for biomedical applications. Hence, it may play an important role in the development of healthcare, mainly for cancer treatment. TiO_2_ has excellent photocatalytic behavior because of its instance photoexcitation which determines the capability to kill cancer cells. The photoexcited activity of TiO_2_ has determined the capability to destroy cancer cells effectively. TiO_2_ possesses a large bandgap energy (∼3 eV), and because of that, it becomes active only under ultraviolet irradiation [26, 27]. Doping with another element has been reported to be one of the most effective methods for turning over new electronic structures and heteroatomic surface structures, consequently resulting in the enhancement of its photocatalysis efficiency in solar light irradiation. TiO_2_ also acts as a functional material for photodegradation as well as photodynamic cancer therapy [28–30].

Reduced graphene oxide (rGO) is an exciting material with excellent thermal stability and electrical conductivity as well as high surface area and thereby has been used in numerous applications, including batteries, photodetectors, and sensors [31–33]. Metal and ferrite-based rGO materials have increased the ability of rGO material toward other fields such as energy harvesting, drug delivery, wastewater contaminants removal, antibacterial efficiency, and cancer therapy [34, 35].

Several techniques have been adopted for the synthesis of metal ferrite NPs such as electrochemical, chemical, photochemical, and biological methods [36, 37], wherein some are still carrying on use (with help) of hazardous chemicals and complex reaction conditions. Chemical synthesis methods are the most common to synthesize nanoparticles explicitly due to the short reaction time [38–40], where hazardous chemicals are used as reducing and capping agents that produce environmental pollution. Modern research focuses on the synthesis of metal ferrite NPs using green synthesis because of its eco-friendly nature, simplicity, and low cost. Green synthesis draws the great inspiration from nature plants and microorganisms of yeast, bacteria, and fungi [41–43]. The different parts of plant extracts help to synthesize metal ferrites via different chemical methods as a result reducing the risk of hazardous chemicals. The plant extracts can produce a variety of metabolites with amino acids, phenols, vitamins, polysaccharides, and carbohydrates, which can react as reducing and capping agents in addition to serving as fuel. Nowadays, many researches report on the green synthesis of NPs using plant extracts as stabilizing and reducing agents [44, 45], even though this method takes a longer reaction time than chemical synthesis. Microwave irradiation method helps to overcome this problem and offers several advantages, including short reaction time under controlled environment, high purity product, uniform heating, and high yield [46, 47]. The rapid uniform heat production and the energy can interfere the sample with the molecular motion. The generated enormous heat originates through molecular collisions, while its uniform distribution is transferred to the interior of the material resulting in an exothermic reaction with the evolution of gases to produce nanoparticles [48].

*Pedalium murex* is of the family of Pedaliaceae generally known as Gokshru presented throughout the world especially in India, Pakistan, and Sri Lanka. This plant was used as medicine in India, including analgesic, kidney stone treatment, stomachache, urinary retention, and intestinal infections. The leaves and roots extract of this plant are used as an aphrodisiac, antimicrobial, antioxidant, antibacterial, and neuroprotection [49–53].

Numerous methods have been adopted to prepare core@shell NPs such as electrostatic layer-by-layer, hydrolysis, and hydrothermal methods. Nevertheless, the sol-gel method is the most frequently used because of its simplicity and low cost in preparing different and stable core@shell NPs [54, 55]. In this paper, NiFe_2_O_4_@TiO_2_@rGO magnetic core@shell NPs have been successfully prepared by the sol-gel method, considering NiFe_2_O_4_ as a core and the fact that its outer layer is functionalized simultaneously by TiO_2_ and rGO. Structural, morphological, optical, and magnetic properties have been investigated. The synthesized samples have been studied in hyperthermia and antibacterial treatment as well as environmental pollution of TC degradation in water bodies.

## 2. Experimental Part

### 2.1. Materials

The chemicals Ni (NO_3_)_2_.6H_2_O, Fe (NO_3_)_3_.9H_2_O, tetrahydrofuran (Merck), ascorbic acid, titanium isopropoxide [C_12_H_28_O_4_Ti] purchased from Merck, graphite (Merck), conc. H_2_SO_4_ (Merck), and KMnO_4_ (Merck) are of analytical grade. *Pedalium murex* leaf extract is used as a reducing agent. Deionized water (DIW) has been used during all preparation stages.

### 2.2. Preparation of Plant Extract

5 g portion of *Pedalium murex* leaves was thoroughly washed, then mixed with 30 ml of DIW, and then subjected to magnetic stirring for about 1 hour at room temperature to get a homogenous solution, followed by filtration to finally obtain the extract.

### 2.3. Materials' Synthesis

#### 2.3.1. NiFe_2_O_4_-CHM NPs

In the molar ratio of 1 : 2, nickel nitrate (Ni(NO_3_)_2_·6H_2_O) and ferric nitrate (Fe(NO_3_)_3_·9H_2_O) were dissolved separately in the DIW, mixed, then kept under vigorous stirring for several hours at room temperature in order to obtain a homogenous clear solution. After that, the *Pedalium murex* leaf extract was added slowly in drops to the above solution for several hours until a very clear solution was obtained. The latter was introduced into a silica crucible and then subjected to irradiation at a frequency of 2.54 GHz at 850 W output power for 12 minutes using a domestic microwave oven. The obtained solid powder was subsequently dried in a hot air oven at 100°C for 1 h, grounded in a mortar pestle, and washed with ethanol.

#### 2.3.2. Core@Shell NiFe_2_O_4_@TiO_2_ NPs

NiFe_2_O_4_ 0.043 mol was added to 150 ml of tetrahydrofuran under continuous stirring, and then ascorbic acid was added to the colloidal solution produced after 2 hours. 0.1 mol of titanium isopropoxide [C_12_H_28_O_4_Ti] (TTIP) was added dropwise under rigorous stirring. At 25°C for 48 h, the resulting colloidal product was dried and then calcined at 500°C for 3 h in a muffle furnace to generate an anatase shell. NiFe_2_O_4_@TiO_2_ core@shell was obtained.

#### 2.3.3. Preparation of Graphene Oxide (GO)

Using the modified Hummer methods, GO was synthesized. Under vigorous stirring in an ice bath, 1 g of natural graphite powder was added to 30 ml conc. H_2_SO_4_, and the temperature was maintained at 20°C. Then, 6 g of KMnO_4_ was added slowly with regular intervals of time for 16 h, and the as-obtained mixture was stirred at room temperature. About 500 ml of ice-cold water was slowly added to the mixture for the endorsement of the complete oxidation, where the color changed from murky brown to dark brown. For the change of color from dark brown to yellowish-brown, 5 ml of 30% H_2_O_2_ was added. With 200 ml of 1 : 10 HCl aqueous solution, the graphite oxide solution was washed numerous times with DIW to remove any residual metal ions. Finally, the graphite oxide solution in DIW was subjected to sonication, centrifugation at 6000 rpm, and ultrasonication for 30 min in order to remove the multilayered graphite oxide.

#### 2.3.4. Core@Shell NiFe2O4@TiO2@rGO NPs

In the previously prepared NiFe_2_O_4_@TiO_2_ homogenous solution, the prepared 0.1 mol liquid GO was added slowly dropwise under continuous stirring. For 2 days at room temperature, the obtained colloidal solution was dried and then calcined at 600°C for 3 h in a muffle furnace. Thus, the final powder obtained was NiFe_2_O_4_@TiO_2_@rGO.

### 2.4. Characterizations

The structural analysis by X-ray diffraction (XRD) was performed using a high-resolution Rigaku Ultima IV diffractometer equipped with a CuK*α* radiation source (*λ* = 1.5418 Å). Rietveld refinement was analyzed by the PDXL program. FTIR was studied using a 4000–400 cm^−1^ range of Perkin Elmer infrared spectrophotometer. HR-SEM analysis was carried out by VEGA 3 TESCAN, and EDX was fiend out by Bruker Nano. Morphological observations were checked by high-resolution transmission electron microscopy (TEM) using JEOL 2000 EX operating at 200 kV. Raman spectra were recorded under 633 nm laser excitation using a Raman spectrometer. Magnetic measurements were performed at room temperature by using a vibrating sample magnetometer (VSM) PMC Micro Mag 3900 equipped with 1 Tesla magnet and a resolution of 0.5 *μ*emu.

### 2.5. Self-Heating Characteristics

Self-heating capacity of the as-prepared NPs was tested using Magnetherm from Nanotherics Ltd. The temperature versus time curves for all samples was recorded under an alternating current (AC) magnetic field of 32.5 mT and a frequency of 622 kHz. For each sample, three different concentrations were applied (5, 10, and 20 mg/mL). All powders were dissolved in distilled water by using pulsed ultrasound vibrations for 10 min. The experiment was conducted starting from the room temperature for all samples (room temperature).

To further evaluate the self-heating characteristics of the as-prepared nanoparticles, the specific absorption rate (SAR) was calculated by using the following equation [50]:

(1) $$\begin{array}{c} \text{SAR}=C\times \frac{\Delta T}{\Delta t}\times \frac{m_{w}}{m_{s}}, \end{array}$$

where *C* is the specific heat capacity of water (4.180 (J/(g.C))); ∆*T*/∆*t* represents the initial slope of the temperature change within the first minute; *m*_*s*_ and *m*_*w*_ are the mass of the sample and water, respectively.

### 2.6. Sonocatalytic, Photocatalytic, and Sonophotocatalytic Activities

The sonocatalytic, visible photocatalytic, and sonophotocatalytic degradation of TC antibiotics was investigated by using NiFe_2_O_4_ (NiF), NiFe_2_O_4_@TiO_2_ (NiT), and NiFe_2_O_4_@TiO_2_@rGO (NiTG) core@shell nanostructures as catalysts. The 30 mg of the catalyst was dissolved separately in the 100 ml of 20 mg/L TC. Prior to the degradation experiments, the mixture was stirred in a dark condition for 30 min to attain the adsorption and desorption equilibrium of TC on the photocatalyst.

In the sonocatalytic degradation, the reaction mixture was taken in the beaker, and then the 2/3rd portion was immersed in the ultrasonic bath and sonicated with 40 kHz frequency. During the reaction, the ultrasonic bath temperature was maintained at 25 ± 5°C by adding ice cubes and exchanging water frequently. The sample was collected every 15 min for further UV-visible characterization.

In the photocatalytic process, a 150 W Halogen lamp was used as visible light in the degradation of TC. The reaction mixture was introduced into the quartz tube placed at 8 cm from the Halogen lamp. During the degradation, the temperature was controlled with a continuous water flow. During the irradiation reaction, 2 ml of solution was collected and then characterized by using UV-visible spectroscopy to estimate the rate of TC degradation.

In the sonophotocatalytic method, the degradation of TC was carried out with the help of both ultrasonic and visible light irradiation. The reaction mixture was placed in the ultrasonic bath with visible light irradiation, and the exchange of ice water controlled the reaction temperature. Every 15 min, a portion of the solution was collected for UV-visible characterization.

The following equation was used to determine the degradation efficiency:

(2) $$\begin{array}{c} \%=\frac{c_{0}-c_{0}}{c_{0}}, \end{array}$$

where *C*_0_ is the initial concentration of the antibiotic and *C*_*t*_ is its concentration after a certain time.

### 2.7. Antibacterial Studies

The antibacterial activity of the as-prepared NiF, NiFT, and NiFTG nanostructures was tested against both Gram-positive and Gram-negative bacterial strains using the agar diffusion method. The bacterial cells were cultured on solidified nutrient agar in Petri plates, five wells were created by sterile well-borer for each plate, and then the sample was loaded to the corresponding well. After 24 h of incubation at 37°C, the antibacterial activity of the nanoparticles was analyzed by measuring the diameter of the clear area of the inhibition zone around the wells.

## 3. Results and Discussion

### 3.1. X-Ray Diffraction Analysis

**Figure 1:**
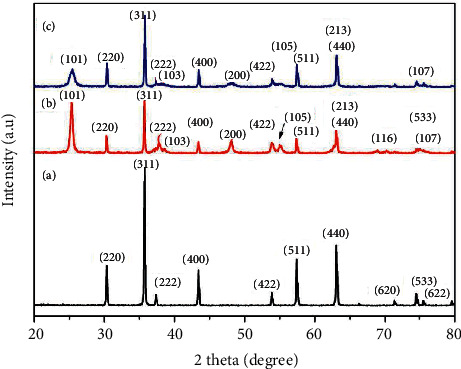
XRD patterns of (a) NiFe_2_O_4_, (b) NiFe_2_O_4_@TiO_2_, and (c) NiFe_2_O_4_@TiO_2_@rGO core@shell nanoparticles.

Figure 1 displays powder X-ray diffraction patterns of the prepared NiFe_2_O_4_, NiFe_2_O_4_@TiO_2,_ and NiFe_2_O_4_@TiO_2_@rGO core-shell NPs. From Figure 1(a), the diffraction peaks located at 30.3°, 35.6°, 37.3°, 43.4°, 53.7°, 57.4°, 63.0°, 71.3°, 74.4°, and 75.5° correspond to the (220), (311), (222), (400), (422), (511), (440), (620), (533), and (622) planes, indexed within the cubic structure of the spinel NiFe_2_O_4_ phase in agreement with the standard JCPDS card no. 00-023-1119. From Figure 1(b), the additional diffraction peaks located at 25.4°, 38.6°, 48.1°, 53.9°, 62.2°, 69.3°, 70.6°, and 74.9° represent the (101), (103), (200), (105), (213), (116), and (107) planes of the tetragonal structure of anatase TiO_2_ phase in agreement with the standard JCPDS card no. 001–0562. It can be noticed that the relative intensity of NiFe_2_O_4_ diffraction peaks decreases due to the formation of the TiO_2_ layer [56]. Figure 1 shows that the main peaks of the NiFe_2_O_4_ did not change when coated with TiO_2_ and rGO. This confirms the stability of the spinel phase of the NiFe_2_O_4_ structure after annealing.

In Figure 1(c), the diffraction peaks of rGO cannot be observed in the recorded XRD pattern. This can be associated with several concurrent effects, including the following: (i) the position rGO (002) plane is very close to the anatase (101) plane at 25°; thereby, overlapping occurs, resulting in lowering the anatase (101) plane intensity [57]; (ii) in X-ray diffraction, peaks' intensity is dependent on the atomic number of the constituents; in this case, rGO (carbon-6) is much lower compared to NiFe_2_O_4_ (nickel-28 and iron-26) as well as TiO_2_ (titanium-22) in addition to (iii) the small amount and amorphous structure of rGO compared to that of NiFe_2_O_4_ (dominant) and TiO_2_ [58]. It is important to highlight that no additional peaks belonging to impurities can be detected within the resolution limit of the X-ray diffractometer, thereby signifying that the as-prepared core@shell samples have a single phase with high purity.

The crystallite size (*D*) of the particles has been determined by the Scherrer formula:

(3) $$\begin{array}{c} D=\frac{0.9\lambda }{\beta \;\cos\;\theta }, \end{array}$$

where *λ* is the wavelength of the X-ray radiation source (1.5418 Å), *β* is the full width at half maximum of the most intense diffraction peak (in radians), *θ* is its corresponding diffraction angle, and 0.9 is a constant for spherical shaped particles. The calculated value of the crystallite size of NiFe_2_O_4_, NiFe_2_O_4_@TiO_2_, and NiFe_2_O_4_@TiO_2_@rGO core-shell NPs is found to be 35, 26, and 23 nm [59]. From Figure 1, it can be observed that the diffraction peaks of NiFe_2_O_4_@TiO_2_ and NiFe_2_O_4_@TiO_2_@rGO become broad compared to that of NiFe_2_O_4_, an indication of crystallite reduction, that is, from 35 to 23 nm [60]. This reduction in crystallite size can be explained as follows: the formation of the TiO_2_ layer and subsequently TiO_2_@rGO double layers onto NiFe_2_O_4_ nanoparticles during the synthesis process inhibited grain growth. It is well known that nanoparticles' formation occurs mainly in 3 stages: nucleation, coalescence, and growth. During the coalescence of NiFe_2_O_4_ nanoparticles already formed, TiO_2_ and then rGO start clustering on their surface, thereby hindering their growth.

### 3.2. Rietveld X-Ray Diffraction Analysis

**Figure 2:**
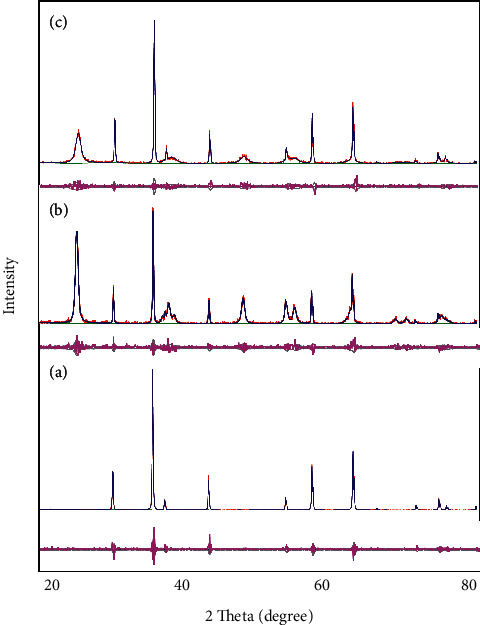
Rietveld image of (a) NiFe_2_O_4_, (b) NiFe_2_O_4_@TiO_2_, and (c) NiFe_2_O_4_@TiO_2_@rGO core@shell nanoparticles.

Figure 2 illustrates the Rietveld refined XRD patterns of NiF, NiT, and NiTG core@shell photonanocatalysts. The upper field data depicts the observed diffraction and calculated patterns, whereas the lower field shows the difference between the observed and calculated diffraction patterns. In Figures 2(a)–2(c), upper field, the diffraction peaks appearing at 29.9°, 35.2°, 37.0°, 42.8°, 53.1°, 56.6°, 62.2°, 73.6°, 74.6°, and 78.7° correspond to the planes (211), (311), (222), (400), (422), (511), (440), (620), (533), and (444) of the cubic spinel structure of NiFe_2_O_4_ phase in agreement with the JCPDS card no. 003–0875. The upper field of Figure 2(b) shows that the additional diffraction peaks located at 25.4°, 38.6°, 48.1°, 53.9°, 62.2°, 69.3°, 70.6°, and 74.9° represent the planes (101), (103), (200), (105), (213), (116), and (107) of anatase tetragonal structure phase, which match well with the JCPDS card no. 001–0562. The diffraction peaks' intensity if Figure 1(a) is higher than that of Figures 2(b) and 2(c) due to the TiO_2_ and rGO shells' formation. The rGO diffraction peaks cannot be detected in Figure 2(c). The (101) peak intensity in Figure 2(c) is smaller than that in Figure 2(b); it may be the overlapping between the rGO (002) and TiO_2_ (101) planes since both are very close to 25°. No additional peaks can be observed, indicating that the as-prepared photonanocatalysts are of high purity.

The Rietveld refinements are performed to determine the crystallite size, microstrain, and lattice parameters.  Table 1 displays the fitting parameters. Both *S* and *χ*_2_ values are statistical findings that indicate how close the experimental pattern is to the calculated one. The *S* value and *χ*_2_ are very close to 1, indicating a very good fit. The goodness of fit value is around 1.179, 1.166, and 1.175 for NiF, NiT, and NiTG, respectively, which shows the good agreement between the observed and calculated XRD patterns [61].

The crystallite size as obtained from the Rietveld refinements is found to be in the same range, that is, 57, 52, and 47 nm for NiF, NiT, and NiTG, respectively. Similarly, the value of the microstrain varies slightly, that is, 0.057, 0.035, and 0.084% for NiF, NiT, and NiTG, respectively. The lattice parameters of TiO_2_ are found to be 3.787 and 3.790 Å.

### 3.3. Fourier Transformed Red Analysis

**Figure 3:**
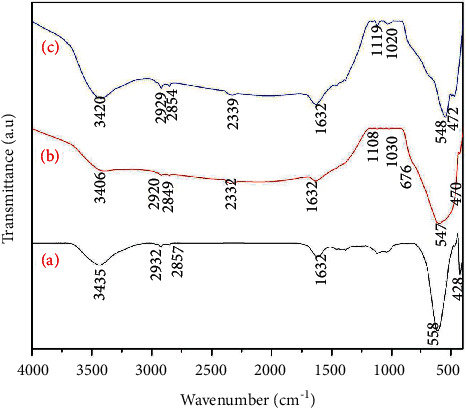
FTIR patterns of (a) NiFe_2_O_4_, (b) NiFe_2_O_4_@TiO_2_, and (c) NiFe_2_O_4_@TiO_2_@rGO core@shell nanoparticles.

Figure 3 shows the vibration of the absorption bands of the as-prepared core-shell NPs in the lattice within the range 400–4000 cm^−1^. Consistently, two strong high-frequency bands located in the ranges of 540–600 cm^−1^ and 400–450 cm^−1^ correspond to tetrahedral and octahedral vibration sites of Ni-O and Fe-O complexes, respectively. The strong high-frequency peaks located at 558 cm^−1^ and 428 cm^−1^ can be assigned to the tetrahedral Ni-O stretching vibration (Ni^2+^) and Fe-O stretching vibration (Fe^3+^), respectively [62], thereby confirming the formation of the spinel NiFe_2_O_4_ phase. The broad peak observed at 900–570 cm^−1^ corresponds to the Ti-O-Ti bending of the anatase phase [63]. The vibration of bands centered at 1650–1550 cm^−1^ and 3600–3300 cm^−1^ refers to the characteristic band of the –OH group, indicating the absorbed hydroxyl groups at the surface of nanoparticles [64]. The common dissolved CO_2_ antisymmetric vibrational band is observed at 2330–2350 cm^−1^. The functional group associated with rGO is located at 1632 cm^−1^, 1020 cm^−1^, and 1116 cm^−1^, ascribed to the vibration modes of C = O, C-O-C, and C-OH, respectively [65]. Figure 3(c) indicated that the oxygen-containing functional group peak intensity is very small, hence demonstrating the reduction of GO to rGO [66]. Finally, the FTIR spectra of the as-prepared core-shell nanoparticles exhibit typical characteristic bands of NiFe_2_O_4_, TiO_2_, and rGO.

### 3.4. Diffuse Reflectance Spectroscopy Analysis

**Figure 4:**
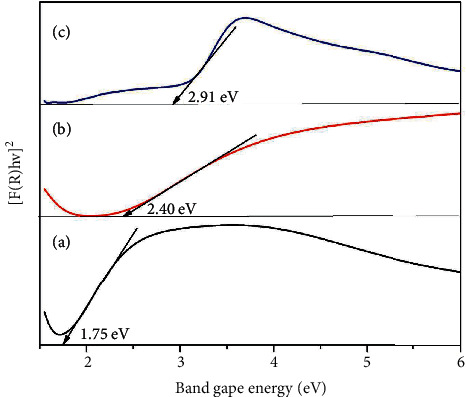
DRS patterns of (a) NiFe_2_O_4_, (b) NiFe_2_O_4_@TiO_2_, and (c) NiFe_2_O_4_@TiO_2_@rGO core@shell nanoparticles.

Figure 4 illustrates the optical properties of NiF, NiT, and NiTG core@shell NPs through UV-visible DRS. The optical bandgap energy value has been determined by means of the Kubelka–Munk function. The graph represents [*F*(*R*)hv]^2^ versus hv plots, where *F*(*R*) is denoted as Kubelka function *F*(*R*) = (1-*R*)^2^/2*R* and *R* is denoted as reflectance in UV-visible spectra. Figure 4 shows that the bandgap values are 1.7, 2.4, and 2.9 eV for NiF, NiT, and NiTG, respectively. As reported by the formula *λ* = 1240/*Eg*, the respective core@shell nanophotocatalysis wavelength absorption is found to be 729, 516, and 427 nm for NiF, NiT, and NiTG [67]. Normally, TiO_2_ has a bandgap energy of around 3 eV, whereas NiT shows an energy bandgap of 2.9 eV (Figure 4(b)). This reduction in the energy band gap can be attributed to the formation of the TiO_2_ layer onto the surface of NiFe_2_O_4_ nanoparticles. This shell formation induces a redshift, as a consequence narrowing the electronic structure level between valence and conduction bands and enhancing the absorption of visible light. The formation of the rGO shell onto the lattice of NiT showed 2.9 eV and gap energy, which will also have the visible light absorption ability. The DRS study indicates that NiTG core@shell NPs possess a good photocatalytic activity and then pure individual compounds.

### 3.5. SEM Observations

**Figure 5:**
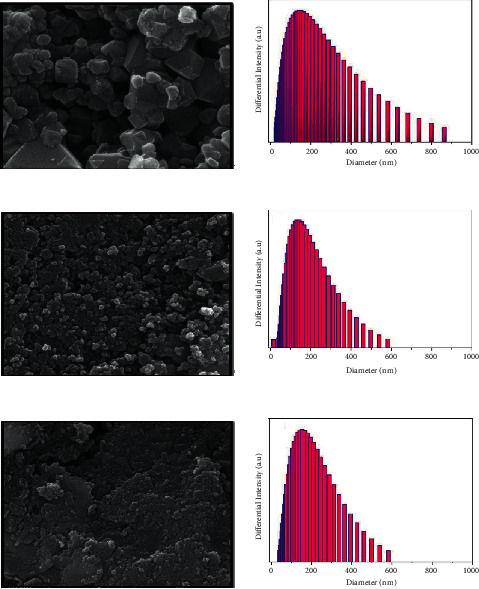
HR-SEM and DLS image of (a and d) NiFe_2_O_4_, (b and e) NiFe_2_O_4_@TiO_2_, and (c and f) NiFe_2_O_4_@TiO_2_@rGO core@shell nanoparticles.

Scanning electron microscopy analysis was carried out to characterize the microstructure of the as-prepared nanoparticles; see Figure 5. SEM image of pure NiFe_2_O_4_ powder (Figure 5(a)) reveals the formation of irregular spherical-like particles at the nanoscale with the tendency to agglomeration due to the interaction between the magnetic dipoles of NiFe_2_O4 NPs. The average diameter of pure NiFe_2_O_4_ NPs is in the range of 70–80 nm.  Figure 5(b) shows small spherical nanostructures of TiO_2_ uniformly decorating pure NiFe_2_O_4_ core NPs, with an average layer thickness of 7–8 nm. For NiFe_2_O_4_@TiO_2_@rGO (Figure 5(c)), both TiO_2_ and rGO layers are loaded onto the surface of pure NiFe_2_O_4_ core, with typical small spherical layers of an exfoliated nanostructure. The average diameter of the rGO layer is about 5–10 nm. The bonding between NiFe_2_O_4_ with TiO_2_ and then TiO_2_ and rGO onto NiFe_2_O_4_ will facilitate transfer of electrons charge separation [68]. SEM observations indicate that the average size of the nanoparticles is larger when compared with the crystallite size obtained by XRD analysis. This is caused due to the high tendency of nanoparticles for agglomeration in the form of *µ*m sized particles [69].

### 3.6. Particle Size Distribution

The average particle size distribution of core@shell nanoparticles has been confirmed by dynamic light scattering (DLS) analysis. Figures 5(d)–5(f) show that the average particle size of NiFe_2_O_4_, NiFe_2_O_4_@TiO_2_, and NiFe_2_O_4_@TiO_2_@rGO is 141.6, 141.6, and 153.2 nm, respectively. The standard deviation of NiFe_2_O_4_ is 106, NiFe_2_O_4_@TiO_2_ is 102.8, and NiFe_2_O_4_@TiO_2_@rGO is 106.7. The mean particle size values obtained by DLS results are also different from that of HR-SEM, which may be due to the salvation properties step carried out in the DLS studies. The DLS determines the particle size distribution of the sample only in the solvated state, whereby there would be solvent molecules associated with the core@shell nanoparticles [70]. The dry state of the powdered samples is only analyzed by HR-SEM.

### 3.7. Energy Dispersive X-Ray Analysis

**Figure 6:**
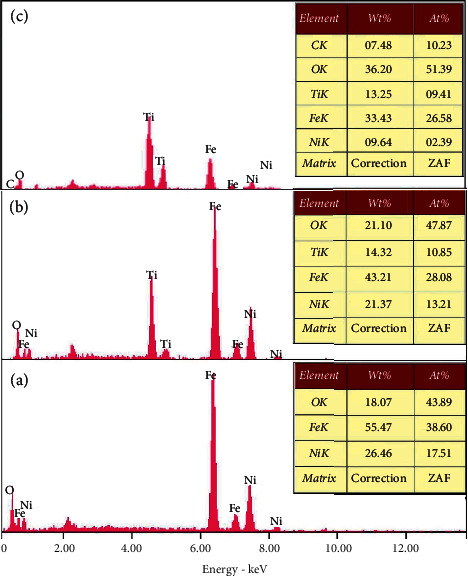
EDX image of (a) NiFe_2_O_4_, (b) NiFe_2_O_4_@TiO_2_, and (c) NiFe_2_O_4_@TiO_2_@rGO core@shell nanoparticles.

The chemical composition of the as-prepared NiF, NiT, and NiTG core@shell NPs has been determined by energy dispersive X-ray (EDX) analysis; see Figure 6. The observed peaks in Figure 6(a) for the NiF sample correspond to nickel, iron, and oxygen elements, which confirm once again the formation of the nickel ferrite phase. Figure 6(b) shows similar elements but with the appearance of titanium peaks, which indicates the formation of the TiO_2_ layer onto NiFe_2_O_4_ core NPs. For the NiTG sample (Figure 6(c)), carbon peaks appear in addition to Ni, Fe, O, and Ti elements, which manifests the formation of NiTG core@shell NPs. The EDX elemental analysis indicates that only Ni, Fe, O, Ti, and C are present, hence confirming the purity and formation of the core@shell NPs.

### 3.8. HRTEM Analysis

**Figure 7:**
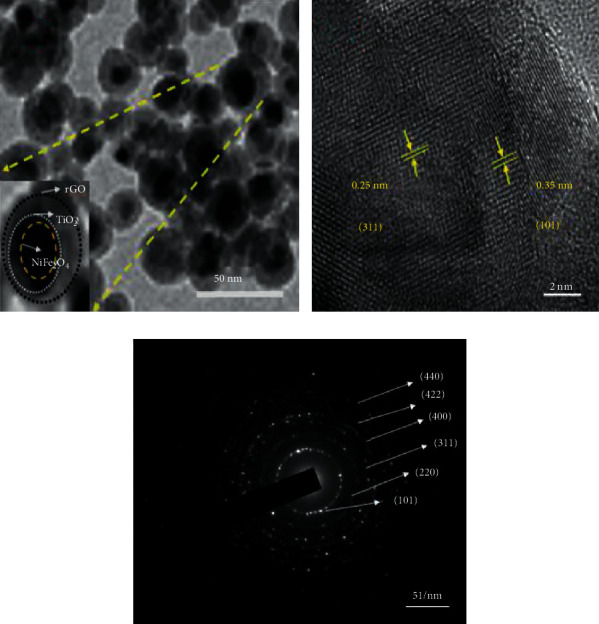
HRTEM image of NiFe_2_O_4_@TiO_2_@rGO core@shell nanoparticles.

The morphology of NiFe_2_O_4_@TiO_2_@rGO core@shell NPs has been further investigated by HRTEM analysis.  Figure 7(a) shows a relative tendency to agglomeration of irregular spherical particles in the nanoscale regime. Because the as-synthesized core@shell NPs possesses different electron absorption ability, NiFe_2_O_4_, TiO_2_, and rGO exhibit different brightness: fine dispersion of dark particle (NiFe_2_O_4_) surrounded by a thin grey layer (TiO_2_) and then a second relatively thick light layer (rGO). The interlattice spacing values are found with d-spaces of 0.25 nm and 0.35 nm, which correspond to the spinel NiFe_2_O_4_ (311) plane and anatase TiO_2_ (101) plane, respectively.  Figure 7(b) displays the SAED (selected area electron diffraction) image of diffraction rings from inside to outside, which can be indexed as (101) plane of anatase TiO_2_ phase and (220), (311), (400), (422), (511), and (440) planes of spinel phase NiFe_2_O_4_, respectively. This SAED pattern confirms that the NiFe_2_O_4_@TiO_2_@rGO core@shell nanostructures are polycrystalline in nature, which corroborates X-ray diffraction analysis.

### 3.9. Magnetic Measurements

**Figure 8:**
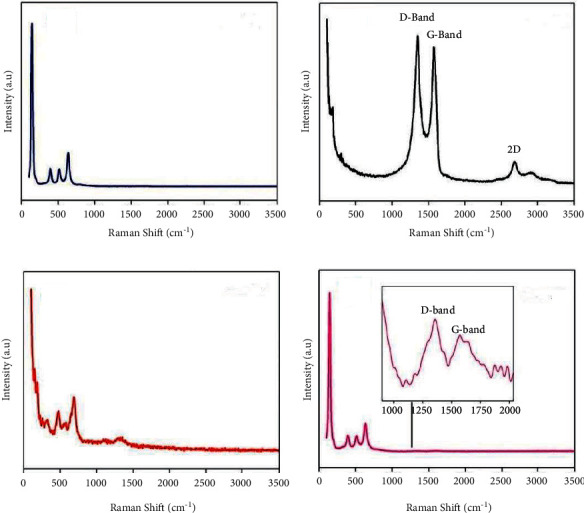
Raman spectra of (a) TiO_2_, (b) rGO, (c) NiFe2O4, and (d) NiFe2O4@TiO2@rGO core@shell nanoparticles.

Figure 8 illustrates the magnetization-field (M-H) curves of the as-prepared NPs recorded at room temperature. It can be observed that all samples exhibit a ferromagnetic behavior with a relatively low hysteresis loop, typical of the soft magnet. However, the magnetic properties (saturation magnetization, remanence, and coercivity) of the core NiFe_2_O_4_ are found to be strongly affected by the coating shells (TiO_2_, rGO); see Table 2. It can be noticed that the saturation magnetization value of NiFe_2_O_4_@TiO_2_ (16 memu/g) and NiFe_2_O_4_@TiO_2_@rGO (15 memu/g) core@shell NPs decreases by almost three times compared to that of NiFe_2_O_4_ (45 memu/g) NPs [62]. Usually, the saturation magnetization value of the sample mainly depends on the amount of magnetic component *M*_*s*_ = *φm*_*s*_, where *φ* and *m*_*s*_ represent the magnetic particles volume fraction and a single magnetic particle saturation magnetization, respectively. The magnetic component amount present in NiFe_2_O_4_@TiO_2_ and NiFe_2_O_4_@TiO_2_@rGO is less than that of NiFe_2_O_4_ NPs [63]. The observed decrease in the saturation magnetization of the spinel ferrite NiFe_2_O_4_ phases is explicitly related to the intrinsic nonmagnetic nature of TiO_2_ and rGO shells. Asha D Patil et al. studied the magnetic property of TiO_2_-doped nanocrystalline Ni-Cu-Zn ferrites, which also exhibited a decrease in saturation magnetization value with the increase in the amount of TiO_2_ in Ni-Cu-Zn ferrites. Similarly, to the saturation magnetization, the remanence of both NiFe_2_O_4_@TiO_2_ and NiFe_2_O_4_@TiO_2_@rGO NPs decreases due as well to the effect nonmagnetic (TiO_2_ and rGO) layers formed onto the surface of magnetic NiFe_2_O_4_ NPs. However, the coercivity is found to increase gradually from 65 to 74 and 80 Oe for NiFe_2_O_4_, NiFe_2_O_4_@TiO_2_@rGO, and NiFe_2_O_4_@TiO_2_@rGO NPs, respectively. The TiO_2_ and rGO shell formation induces an increase in the magnetic particle space interval, which results in an increase in coercivity [71]. Also, the calcination at the relatively high temperature promotes significantly the disorder in the arrangement of the magnetic moment of local atoms within the crystal lattice, consequently resulting in the linear increase of coercivity and the saturation magnetization, in addition to the inverse dependence of particle size and coercivity as reported in the literature [65, 72].

### 3.10. Raman Spectroscopy

**Figure 9:**
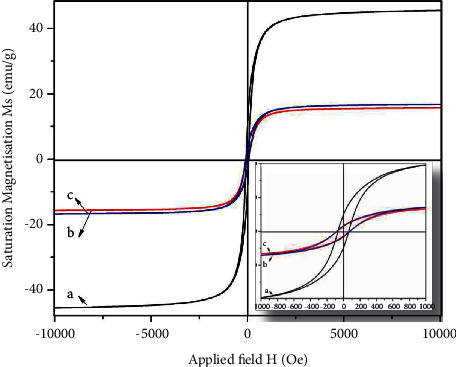
M-H curves of (a) NiFe_2_O_4_, (b) NiFe_2_O_4_@TiO_2_, and (c) NiFe_2_O_4_@TiO_2_@rGO core@shell nanoparticles.

Raman spectroscopy is mainly used to identify the structural defects and disorders present in carbon and carbon-based materials. Figure 8 shows the Raman spectrum of TiO_2_, rGO, NiFe_2_O_4_, and NiFe_2_O_4_@TiO_2_@rGO. As shown in Figure 8(a), anatase TiO_2_ Raman peaks appear at 143, 394, 516, and 632 cm^−1^ corresponding to *Eg*_(1)_, *B*_*1g*_, *A*_*1*_*g* + *B*_*1*_*g*_(2)_, and *Eg*_(2)_ modes. The pure rGO Raman peak of the D-band and G-band appear at 1321 and 1593 cm^−1^ in Figure 9(b). The Raman spectrum of pure NiFe_2_O_4_ located at 259, 310, 463, 546 cm^−1^, and 999 cm^−1^ correlates to *T*_*2g* (1)_, *Eg*, *T*_*2*_*g*_(2)_, *T*_*2*_*g*_(3)_, and *A*_*1*_*g*. In Figure 8(c), the four high intense Raman peaks appearing at 143, 394, 516 cm^−1^, and 632 cm^−1^ can be ascribed to the anatase TiO_2_ phase *Eg*_(1)_, *B*_*1*_*g*, *A*_*1*_*g*+*B*_*1*_*g*_(2)_, and *Eg*_(2)_ modes, respectively [68]. In Figure 8(c), the rGO D-band and G-band of NiFe_2_O_4_@TiO_2_@rGO are observed at 447 and 487 cm^−1^, respectively. The sp^2^ vibration-bonded carbon atoms are denoted as G-band and the carbon materials of carbon atoms vibration with dangling bonds which is indicated as D-band. These G- and D-bands help to know the inadequacy and structural disordered in carbon and carbon-based compounds. The quantity of defects in the graphitic compound is characterized by *I*_*D*_/*I*_*G*_ intensity ratio. The lower value (below 1) represents a degree of GO higher due to the high sp^2^ hybrid carbon atoms. The higher value of *I*_*D*_/*I*_*G*_ (above 1) manifests a higher structural disorder upon the reduction of GO [60]. In Figure S1 (in Supplementary Materials), the intensity *I*_*D*_/*I*_*G*_ ratio value of NiFe_2_O_4_@TiO_2_@rGO core@shell NPs is found to be 1.04, confirming the successful reduction of GO during the core@shell thermal formation. The Raman peak of NiFe_2_O_4_ is unable to find out in Figure 8(d) due to the high-intensity peaks of TiO_2_ overlapping the NiFe_2_O_4_ Raman peaks.

### 3.11. Hyperthermia Application

The generation of heat from the as-synthesized magnetic core@shell nanoparticles in an AC magnetic field for the application of hyperthermia can be examined by the variation of temperature in a function of measured time. The curves of temperature varied with time for pure NiFe_2_O_4_, NiFe_2_O_4_@TiO_2_, and NiFe_2_O_4_@TiO_2_@rGO core@shall nanoparticles with the help of AC magnetic field of 32.5 mT at a 622 kHz frequency. Hence, in the presence of an AC magnetic field, there are four possible mechanisms to explain the temperature increase (Neel relaxation, Eddy current, hysteresis magnetic field, and Brownian relaxation). In the case of our nanoparticles, the temperature rise is due to the particles spinning due to the AC magnetic field. The energy deposition in this case is related to Neel and Brownian relaxations, which are the most dominant in nanoscale [69]. Overall, all the tested samples manifested a considerable heat emission when AC magnetic field was applied.

**Figure 10:**
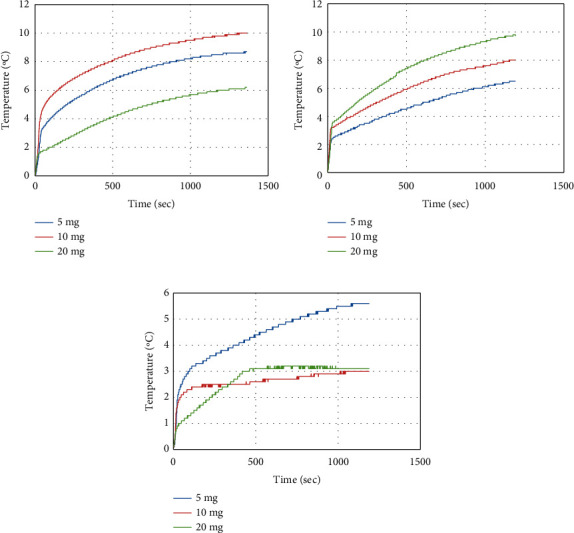
Hyperthermia heating efficiency of (a) NiFe_2_O_4_, (b) NiFe_2_O_4_@TiO_2_, and (c) NiFe_2_O_4_@TiO_2_@rGO core@shell nanoparticles.

Figures 10(a)–10(c) show the results of temperature rise of prepared samples at concentrations of 5, 10, and 20 mg/mL. All heating curves demonstrate the same behavior of temperature increase. In the first minute, a rapid temperature increase occurred and then continued to increase gradually and in an exponential manner. The rapid increase in temperature is due to the rearrangement of particles when the AC magnetic field was applied, and then the next gradual temperature increase could be associated with the hysteresis loss due to the ferromagnetic nature of the as-prepared samples. After certain times, the temperature' curves start to flatten. This might be explained by the low dispersion of the NPs in the solution as particles tend to gradually accumulate in the sample holder base lowering heat dissipation ability.

Furthermore, to evaluate the self-heating efficiency, the specific absorption rates (SAR) were calculated for the samples. The SAR explains the heating effect created per unit mass of the sample. It depends on the initial slope (the 1st minute) of the temperature-time curve as well as the concentration of the sample (equation (1)) [73]. In Table 3, the SAR values are shown along with the XRD crystallite size and magnetization. The temperature rise in degrees is also recorded for 20 minutes. It is essential to consider the rise of temperature degrees as the living cell (at 37°C) can be destroyed at a temperature range of 42 to 47°C for a period of time (10 to 20 mins) depending on the type of treated tissue [74, 75].

Table 3 shows the variation of SAR values of the samples as well as the temperature change depending on the concentration of the sample and also on the coatings applied. NiFe_2_O_4_ experienced the highest SAR value of 58.2 W/g at the lowest concentration of 5 mg/mL. Higher concentrations of NiFe_2_O_4_ (10 and 20 mg/mL) lowered the calculated SAR value; however, at a concentration of 10 mg/mL, the rise in the temperature reached 10°C in only 20 mins. At 10 mg/mL concentration, NiFe_2_O_4_ was dispersed more in the solution giving higher heat dissipation ability. More concentration (20 mg/mL) may lead to agglomeration of NPs and hence reduce their self-heating effect. NiFe_2_O_4_@TiO_2_ NPs also showed considerable SAR values at a maximum of 18 W/g at 10 mg/mL. Lower heating ability is expected for the coated NiFe_2_O_4_@TiO_2_ samples as the saturation magnetization reduces from 45 to 15 emu/g, which leads to less magnetic induced self-heating ability. When rGO was introduced to the samples, a considerable decrease was noticed in the slope and the increased temperature, especially when increasing the concentrations. At a concentration of 5 mg/mL, NiFe_2_O_4_@TiO_2_@rGo samples have shown an initial temperature change that is higher than that of the NiFe_2_O_4_@TiO_2_ samples; this also led to a higher SAR value of 40.5 W/g in comparison with 31 W/g for the NiFe_2_O_4_@TiO_2_. This can be explained by the formation of powerful anisotropy and reduced aggregation of the TiO_2_ and rGO shells formed on the NiFe_2_O_4_ nanoparticles, which helps the NiFe_2_O_4_@TiO_2_@rGO heating effect, which is better than NiFe_2_O_4_ and NiFe_2_O_4_@TiO_2_ [76, 77]. However, at higher concentrations (10 and 20 mg/mL), NiFe_2_O_4_@TiO_2_@rGO samples tend to be less dispersive in distilled water solution and to accommodate quickly at the bottom of the sample holder, which may explain the low self-heating parameters as shown in Table 3.

Table 4 shows the comparative values of the as-prepared core@shell nanoparticles. Overall, our prepared samples have shown significant SAR values when compared with other values calculated by a previously published work in the literature, taking into consideration the properties of the nanoparticles as well as the self-heating experiment parameters (magnetic field strength and frequency). Table 4 displays a comparison of the SAR values obtained in this study with some recent similar works. Manoher and Hong have prepared NiFe_2_O_4_ through the solvothermal reflux method, which resulted in very small nanoparticles (9 nm) with a high saturation magnetization of 46.86 emu/g [78]. A very high SAR value (96.86 w/g) was obtained for these NiFe_2_O_4_ samples at a high magnetic field of 294 mT and a frequency of 316 kHz, whereas the combusted NiFe_2_O_4_ samples obtained in our work exhibited a SAR value of 58.2 W/g with only 32.5 mT magnetic field strength. Other ferrites nanoparticles have also shown high SAR values at higher magnetic field strengths such as ZnFe_2_O_4_ and CoFe_2_O_4_ [79, 80]. Other studies that conducted the self-heating experiment at lower magnetic field strength experienced lower SAR values such as autoclave MgFe_2_O_4_ samples, only 20 W/g at 17 mT [81]. For hyperthermia application, it is very important to consider the clinical magnetic field, which is reported to be around 20 mT (Zhang et al., 2019), as well as the temperature rise [9]. High magnetic fields and high-rate temperature rise could cause harmful side effects to the surrounding healthy tissues [82]. It is also essential to mention that the core@shell design gives the particles the potential to be applied as an efficient drug carrier [83]. Our prepared NiFe_2_O_4_@TiO_2_@rGO core-shell NPs have shown considerable self-heating efficiency in terms of temperature change as most of the samples showed a temperature rise of 6 to 10 degrees in 20 min, which is within the hyperthermia therapeutic range [84]. The obtained SAR values at relatively low magnetic field strength have also shown a great potential for further research on hyperthermia and drug delivery.

### 3.12. Sonophotocatalytic Degradation

The TC degradation efficiency of NiF, NiT, and NiTG core@shell photonanoparticles has been assessed by sonocatalysis, photocatalysis, and sonophotocatalytic methods. The TC degradation with respect to time was evaluated by UV-Vis spectroscopy. The UV-Vis spectrum exhibits two main absorption bands at 258–298 nm and 326–378 nm wavelengths corresponding to the presence of tricarbonyl amide and phenolic diketone group resonating, respectively. Initially, the reaction mixture adsorption-desorption equilibrium of catalysis and TC solution was carried out for 30 min under dark condition. No significant antibiotic degradation rate on the catalytic surface of all three photocatalytic NPs has been observed.

**Figure 11:**
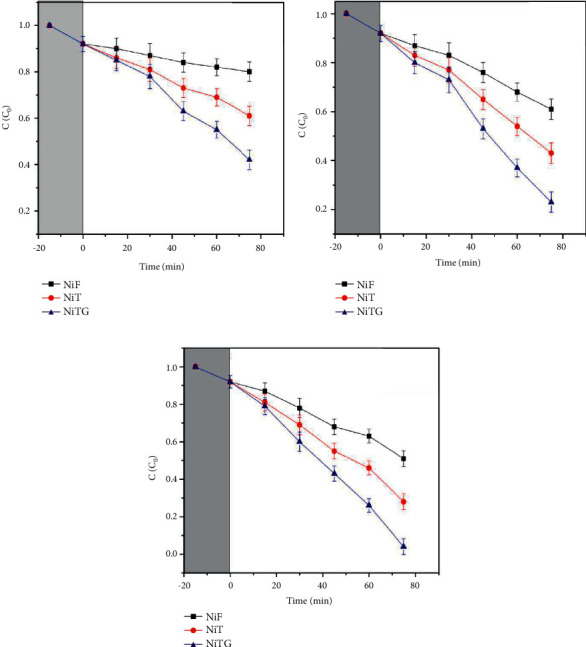
C/C_0_ versus time plot of TC degradation by (a) sonocatalytic; (b) photocatalytic; (c) sonophotocatalytic methods.

Figure 11(a) illustrates the UV-Vis absorbance results of TC after the sonocatalytic degradation using NiF, NiT, and NiTG nanophotocatalysts. In the sonocatalysts process, the ultrasonic irradiation cleans the surface of the catalyst NPs rapidly to enhance the material mass transfer and organic compound accumulation reduction and avoid unwanted byproducts. For a contact time of 75 min, the degradation efficiency of TC increases gradually and significantly in the following order: 20% for NiF, 40% for NiT, and 60% for NiTG.

Figure 11(b) depicts the TC degradation by a visible light photocatalytic method using NiF, NiT, and NiTG nanophotocatalysts. The visible light energy enhances the photocatalytic electron transfer from the valence band to the conduction band, thereby accelerating the degradation of TC. The degradation rate of TC is enhanced significantly from only 40% for NiF to 60% for NiT, and then reaching 80% for NiTG was for a contact time of 75 min.

Figure 11(c) displays the TC degradation by the combined sonophotocatalytic process using NiF, NiT, and NiTG nanophotocatalysts. A similar trend is also observed; the efficiency of TC degradation for a contact time of 75 min is enhanced significantly, achieving 50%, 70%, and 90% for NiF, NiT, and NiTG, respectively.

**Figure 12:**
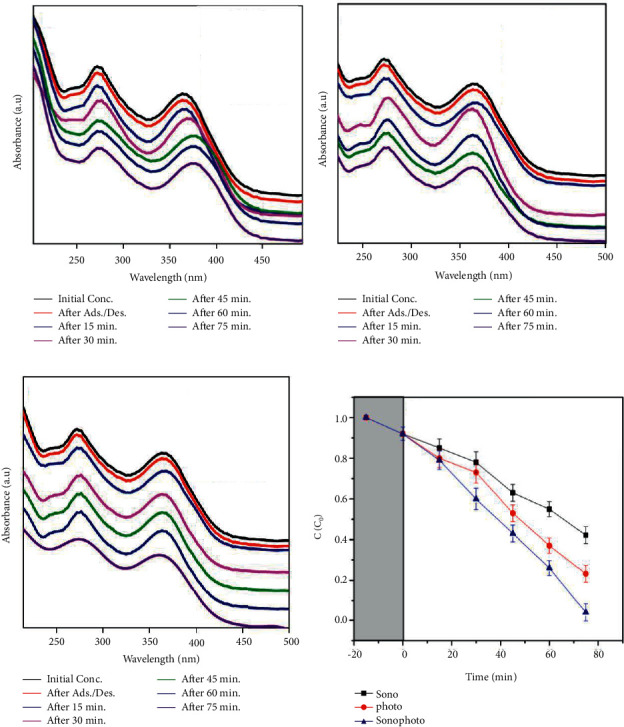
TC degradation by (a) Ni; (b) NiT; (c) NiTG (d) C/C_0_ versus time.

For the three tested TC degradation methods, it is clearly noticed that the ternary NiTG core@shell NPs demonstrate the highest degradation efficiency compared to the binary NiF and NiT core@shell NPs (Figures 12(a)–12(c)). At the same time, comparing the three different photocatalytic methods, the combined sonophotocatalytic process stands as the most efficient compared to sono- and UV-visible photocatalytic methods taken individually (Figure 12(d)).

### 3.13. Tetracycline Degradation Mechanism

**Figure 13:**
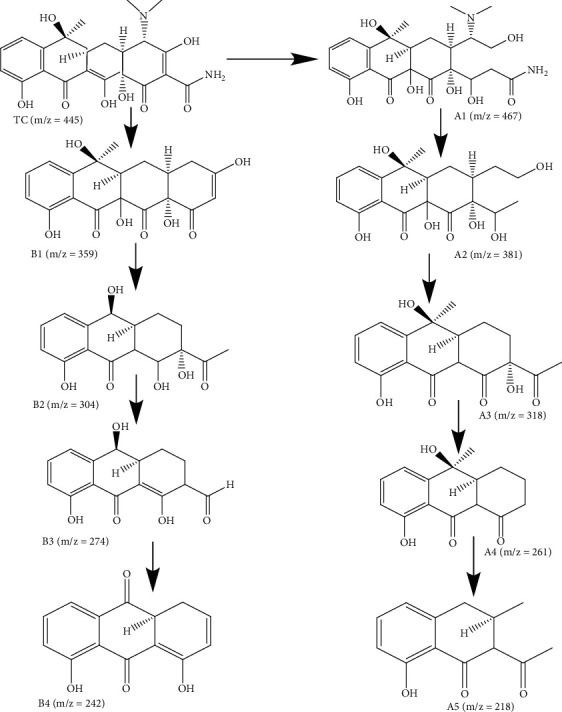
The proposed sonophotocatalytic tetracycline degradation mechanism.

The degradation pathway of TC is analyzed using the LC-MS technique to determine the intermediate products obtained from visible light degradation. The prominent peak of TC is at *m*/*Z* = 445. In general, functional groups such as double bonds, amine, and phenolic groups are degraded due to their relatively high electron density, out of which double bonds have higher reactivity than others and therefore readily undergo hydroxylation to produce primary product 2 (*m*/*Z* = 467) [88]. Due to the weak binding energy of N-C, product 3 (*m*/*Z* = 381) and product 4 (*m*/*Z* = 359) are obtained by N-demethylation and deamidation. Product 4 is demethylated with the removal of CH_2_C(OH) to produce product 6 (*m*/*Z* = 304). Upon further reaction, product 7 (*m*/*Z* = 274) and product 8 (*m*/*Z* = 242) are obtained. Upon oxidation and dealkylation processes, product 3 is converted to product 5 (*m*/*Z* = 318). Upon further reaction progress of dihydroxylation, dealkylation, and ring opening, product 9 (*m*/*Z* = 261) and product 10 (*m*/*Z* = 218) are attained [89]. The determination of the degradation pathway of TC under visible light is shown in Figure 13, and the LC-MS spectra of the derived products are elaborated in more detail in Figure S2 (in Supplementary Materials) [90]. The obtained results of the degradation pathways indicate that, upon further degradation reactions, the TC molecules would oxidize and progressively degrade to small molecules.

The TC degradation by NiFe_2_O_4_@TiO_2_@rGO using visible light achieved 90% in 75 min. The degradation mechanism under visible light is examined to determine the involved pathways. First, TC molecules are adsorbed on the NTG surface by electrostatic attraction and *π* − *π*  stacking. When NTG is irradiated under visible light, the molecules absorb photon energy which surpasses their bandgap energy to produce electron-hole pairs. The electrons (e^−^) on the surface of NTG are transferred from the valence band (VB) to the conduction band (CB), and holes (h^+^) will be produced in the VB. The CB and VB for NiFe_2_O_4_ are found to be 0.62 and 2.91 eV, respectively. The observed CB value is higher than the redox potential of O_2_/O_2_^−^ and hence cannot participate in the reduction process as it is a strong oxidant. Likewise, the holes are at larger oxidation potentials, which favor the oxidation of TC molecules. This is because of the greater value of VB than the redox potential of OH/OH^−^. The holes readily form OH^−^ which undergoes oxidation of H_2_O to form OH radicals. When irradiated by visible light, the TC molecules absorb the photons to undergo self-oxidation transformation. The photogenerated electrons of NiFe_2_O_4_ migrate to the rGO surface, which suppresses the electrons on the CB with the holes on the VB. In addition, h+, OH, and O_2_^−^ combine with the oxidation of TC molecules to degrade tetracycline antibiotics.

### 3.14. Antibacterial Activity Study

**Figure 14:**
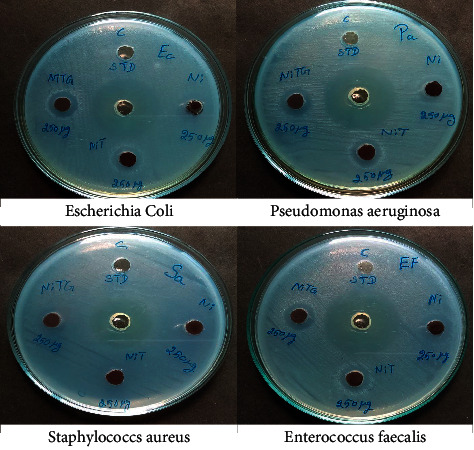
Zone of inhibition against Gram-positive and Gram-negative bacteria in well diffusion method.

The antibacterial activity of the as-prepared NiFe_2_O_4_, NiFe_2_O_4_@TiO_2_, and NiFe_2_O_4_@TiO_2_@rGO core-shell NPs has been investigated by using both Gram-positive and Gram-negative bacterial strains (Figure 14). All tested NPs are smaller in size, so they have a high surface area; thereby, it is expected they can easily reach the bacteria cell to damage the respiration of bacteria and may cause bacterial cell death.  Table 5 indicates that the as-prepared NiTG shows higher antibacterial activity than NiF and NiT. However, all the three samples show higher activity toward Gram-negative bacteria than Gram-positive bacteria. The Gram-negative bacteria have a thin layer wall of peptidoglycan polymer (∼7v-v8 nm), whereas Gram-positive bacteria cells have a thicker wall of multilayer of peptidoglycan (<8 nm). For this reason, the as-prepared core@shell NPs can easily reach the Gram-negative bacteria cell membrane and then Gram-positive bacteria. Besides, NiTG core@shell NPs exhibit good magnetic properties, so they can be easily removed from the solution with an external magnetic field, hence avoiding the environmental pollution. From the antibacterial activity results, the as-prepared NiTG core@shell NPs demonstrate good activity and are environmentally friendly.

## 4. Conclusion

In this paper, multifunctional core@shell of NiFe_2_O_4_@TiO_2_@rGO nanostructures have been successfully synthesized through the green synthesis method and characterized. XRD analysis confirms the formation of single nanocrystalline phases. The TiO_2_ and rGO shell formation onto NiFe_2_O_4_ nanoparticles' surface has been confirmed with interlattice spacing, that is, 0.25 nm for NiFe_2_O_4_ (311) plain and 0.35 nm for TiO_2_ (101) plane by high-resolution transmission electron microscopy. SAED analysis indicates the polycrystalline nature of NiFe_2_O_4_@TiO_2_@rGO core@shell nanoparticles. The reduction of GO present in the prepared NiFe_2_O_4_@TiO_2_@rGO core@shell nanoparticles is evidenced by Raman spectroscopy analysis. VSM analysis establishes the ferromagnetic nature with a noticeable decrease in the saturation magnetization upon the deposition of nonmagnetic layers of TiO_2_ and rGO. A SAR value of 40.5 W/g is obtained for NiFe_2_O_4_@TiO_2_@rGO core@shell nanoparticles at the lowest concentration of 5 mg/mL under 32.5 mT applied AC magnetic field. The obtained SAR values at relatively low magnetic field strength have also shown a great potential for further research in hyperthermia and drug delivery. The TC degradation performed by means of the three methods reveals that NiTG NPs exhibit a higher degradation efficiency than binary NiF and NiT. The measured zone of inhibition indicates that NiTG core@shell NPs have an effective antibacterial activity in Gram-positive and Gram-negative bacterial pathogens. This promotes their potential use for many medical and environmental applications.

## Figures and Tables

**Table 1:** Crystallite size, lattice parameter, and fit parameter of (a) Ni; (b) NiT; (c) NiTG.

Temperature (°C)	Phase composition (%)	Crystallite size (nm)	Microstrain (%)	Lattice parameters (Å)	Fit parameters
NiFe_2_O_4_	NiFe_2_O_4_: 100	57.0	0.057	*a* = *b* = *c* = 8.3435; *α* = *β* = *γ* = 90	*R*_wp_ = 27.12 *R*_e_ = 24.95 *R*_p_ = 15.90
					*S* = 1.0858 *χ*^2^ = 1.1790

NiT	NiFe_2_O_4_: 27.6	52.86	0.0354	*a* = *b* = *c* = 8.3393; *α* = *β* = *γ* = 90	*R*_wp_ = 23.95 *R*_e_ = 22.13 *R*_p_ = 15.39
	TiO2 (anatase): 72.4	17.53	0.227	*a* = *b* = 3.7870, *c* = 9.512, and *α* = *β* = *γ* = 90	*S* = 1.0801 *χ*^2^ = 1.1666

NiTG	NiFe_2_O_4_: 46.7	47.4	0.084	*a* = *b* = *c* = 8.3427; *α* = *β* = *γ* = 90	*R*_wp_ = 25.01 *R*_e_ = 23.02 *R*_p_ = 17.37
	TiO_2_ (anatase): 53	10.02	0.93	*a* = *b* = 3.790; *α* = *β* = *γ* = 90	*S* = 1.0842 *χ*^2^ = 1.1754

**Table 2:** Magnetic properties of core@shell nanoparticles.

Sample	Crystallite size (nm)	Coercivity (Oe)	Remanence (emu/g)	Saturation magnetization (emu/g)
NiFe_2_O_4_	35	65	9	45
NiFe_2_O_4_@TiO_2_	26	74	3	16
NiFe_2_O_4_@TiO_2_@rGO	23	80	2	15

**Table 3:** Hyperthermia heat efficiency and SAR value of core@shell nanoparticles.

Samples	Crystallite size (nm)	Magnetization (emu/g)	Concentration in self-heating experiment (mg)	Initial slope (1^st^ min) (°C/sec)	Max ∆*T* (°C)	SAR (W/g)
NiFe_2_O_4_	35	45	5	0.0696	8.7	58.2
			10	0.0934	10.0	39.0
			20	0.0247	6.2	5.2

NiFe_2_O_4_@TiO_2_	26	15	5	0.0371	6.7	31.0
			10	0.0431	8.2	18.0
			20	0.0645	9.8	13.5

NiFe_2_O_4_@TiO_2_@rGO	23	16	5	0.0485	5.8	40.5
			10	0.0349	3.0	14.6
			20	0.0152	3.2	3.2

**Table 4:** Comparative study of the present work with previous literature.

Sample	Synthesis route	Grain/crystallite size (nm)	Magnetization (emu/g)	Concentration (mg/mL)	Magnetic field (mT)	Frequency (kHz)	SAR (W/g)	Ref.
NiFe_2_O_4_	Microwave irradiation	35	45	5	32.5	622	58.2	This study
NiT	Sol-gel	26	15	5			31	This study
NiTG		23	16	5			40.5	This study
NiFe_2_O_4_	Solvothermal reflux	9	46.86	1	294	316	96.86	[78]
MgFe_2_O_4_	Autoclave	66.8	27.39	10	17	331	20.9	[81]
ZnFe_2_O_4_	Coprecipitation	33	41	5	81	355	125	[79]
ZnFe_2_O_4_	Sol-gel	29	23.59	2	Coil of 75 mm diameter and four turns		105	[85]
CoFe_2_O_4_	Microwave combustion	48	77.29	10	17	331	15.79	[72]
CoFe_2_O_4_	Hydrothermal	6	43	1	120	329	76	[80]
CoFe_2_O_4_	Hydrothermal		58.96				10.63	[86]
CuFe_2_O_4_	Combustion	25.1	29.4	15	17	331	14.63	[65]
PEG_x_Mn_0.5_Zn_0.5_Fe_2-x_O_4_ (*x* = 1.5)	Sol-gel	15.7	30.1	44	180	425	3.5	[87]
*θ*-Fe_3_N@Fe_3_O_4_	Solvothermal process	32	99	10	23	261	90	[91]
MnFe_2_O_4_	Solvothermal method	22	75	2	17	330	—	[92]
HfxFe_3-x_O_4_	Microwave refluxing method	10–30	51.	125	11	479	20	[93]

**Table 5:** Zone of inhibition against Gram-positive and Gram-negative bacterial strains in well diffusion method.

Sample	Gram-positive		Gram-negative	
	*Enterococcus faecalis*	*Staphylococcus aureus*	*Escherichia coli*	*Pseudomonas aeruginosa*
NiFe_2_O_4_	14	10	16	13
NiFe_2_O_4_@TiO_2_	13	12	14	12
NiFe_2_O_4_@TiO_2_@rGO	14	13	15	13

## Data Availability

No data were used to support this study.

## Supplementary Materials

Supplementary Materials Figure S1 shows the Raman spectrum of intensity *I*_*D*_/*I*_*G*_ ratio value of GO (*I*_*D*_/*I*_*G*_ = 0.94) and rGO (*I*_*D*_/*I*_*G*_ = 1.04). This different value confirms the successful reduction of GO. Figure S2 shows the LC-MS spectra for the derived product TC degradation pathway under sonovisible light with the help of NiFe2O4@TiO2@rGO nanoparticles. Table S1 shows the Raman spectrum data values for the reference.
