# Supplementary material for: Multifunctional Core-Shell NiFe2O4 Shield with TiO2/rGO Nanostructures for Biomedical and Environmental Applications
Source: Bioinorg Chem Appl. 2022 May 30;2022:4805490. doi: 10.1155/2022/4805490 (PMC9174006; doi:10.1155/2022/4805490)
Supplement: Supplementary Materials — Figure S1 shows the Raman spectrum of intensity ID/IG ratio value of GO (ID/IG = 0.94) and rGO (ID/IG = 1.04). This different value confirms the successful reduction of GO. Figure S2 shows the LC-MS spectra for the derived product TC degradation pathway under sonovisible light with the help of NiFe2O4@TiO2@rGO nanoparticles. Table S1 shows the Raman spectrum data values for the reference. [file 4805490.f1.docx]

**Supplementary Information**

**Multifunctional Core-Shell NiFe_2_O_4_ Shield with TiO_2_/rGO Nanostructures for Biomedical and Environmental Applications**

R. Esther Nimshi^1^, J. Judith Vijaya^1,*^, B. Al-Najar^2^, L. Hazeem^3^, M. Bououdina^4^, L. John Kennedy^5^, K. Kombaiah^6^, S. Bellucci^7^

^1^ CNR Laboratory, Department of Chemistry, Loyola College, Chennai-34, University of Madras, India

^2^ Department of Physics, College of Science, University of Bahrain, PO Box 32038, Bahrain

^3^ Department of Biology, College of Science, University of Bahrain, PO Box 32038, Bahrain

^4^ Department of Mathematics and Science, Faculty of Humanities and Sciences, Prince Sultan University, Riyadh, Saudi Arabia

^5^ Materials Division, School of Advanced Sciences, Vellore Institute of Technology University, Chennai Campus, Chennai-127, India

^6^ Department of Chemistry, Arul Anandar College, Madurai-625 514, Madurai Kamaraj University, India

^7^ INFN-Laboratori Nazionali di Frascati, Via E. Fermi 40, 00044 Frascati, Italy

* Corresponding author: jjvijaya78@gmail.com (Dr. J. Judith Vijaya)

**
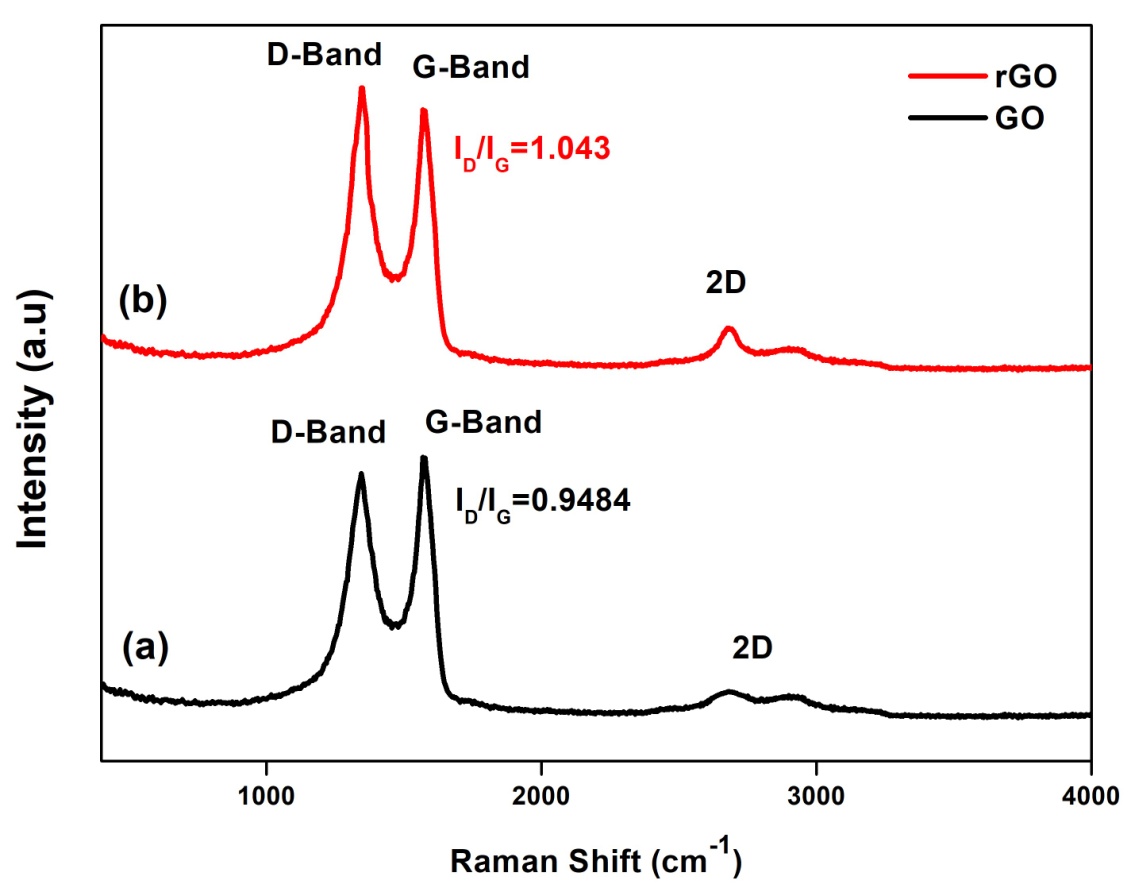
**

**Fig. S1** Raman spectra of GO and rGO nanoparticles


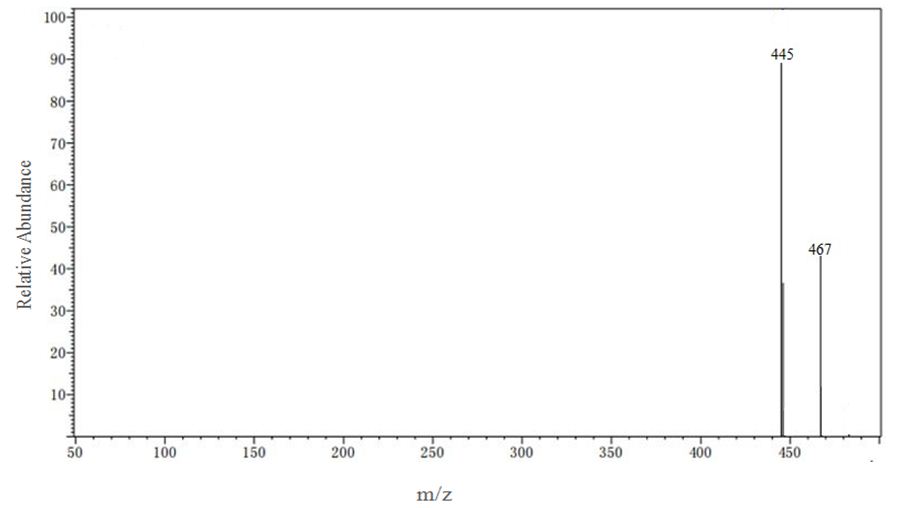

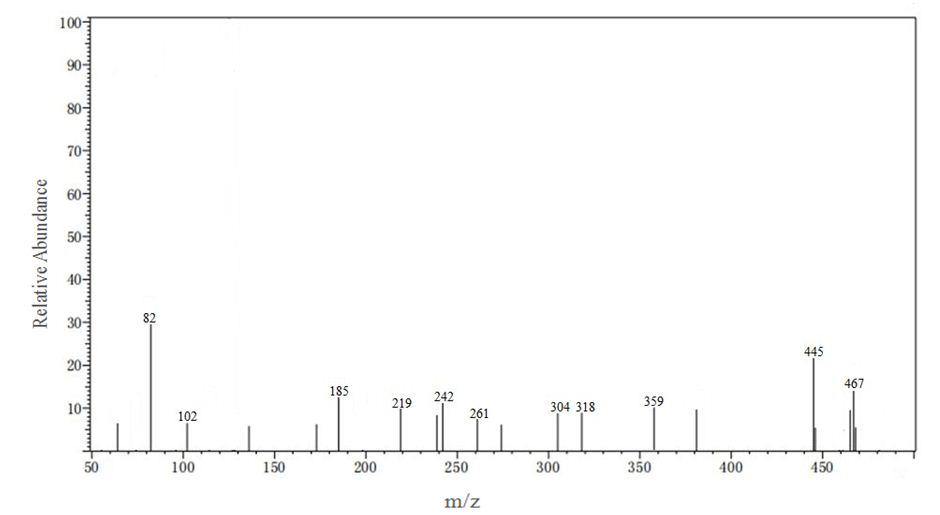

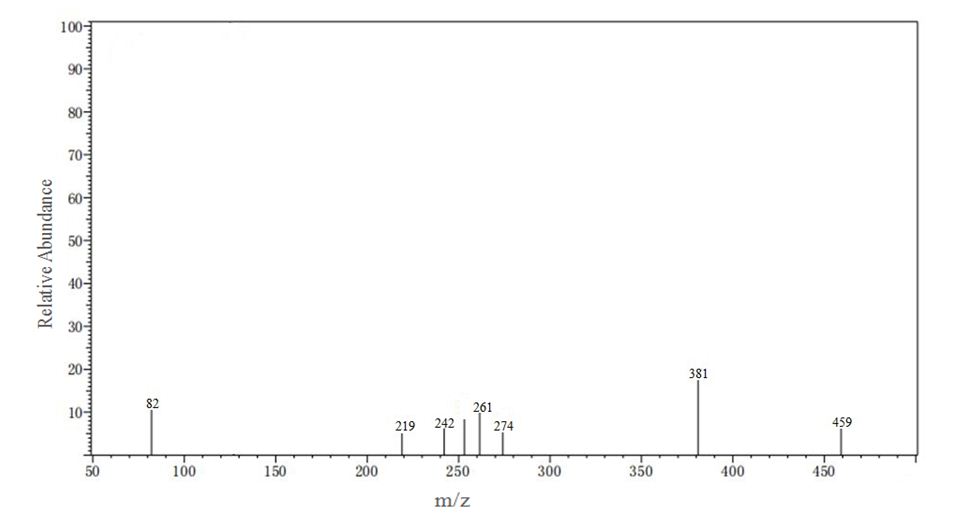


**(a)**

**(b)**

**(c)**

**Fig.S2** LC-MS spectra of the sonophotocatalytic degradation of tetracycline solution at different time interval using NiFe_2_O_4_@TiO_2_@rGO core@shell nanoparticles (a) 0, (b) 30, (c) 75 min

**Table S1** Raman raw data Results

|  | TiO2 | GO | rGO | NF | NTG |
| --- | --- | --- | --- | --- | --- |
| 101.5781 | 3827 | 16004 | 16491 | 2713 | 2795 |
| 106.1459 | 4964 | 12772 | 13259 | 2442 | 3434 |
| 110.7114 | 6164 | 13731 | 14218 | 2052 | 4090 |
| 115.2746 | 8139 | 10996 | 11483 | 1661 | 4983 |
| 119.8356 | 11725 | 9000 | 9487 | 1609 | 6367 |
| 124.3943 | 19818 | 7928 | 8415 | 1592 | 8901 |
| 128.9508 | 31777 | 8576 | 9063 | 1444 | 12743 |
| 133.505 | 47197 | 7715 | 8202 | 1225 | 18387 |
| 138.057 | 60173 | 6915 | 7402 | 1203 | 24105 |
| 142.6068 | 62030 | 7038 | 7525 | 1206 | 28144 |
| 147.1542 | 53469 | 7529 | 8016 | 1404 | 28503 |
| 151.6995 | 39635 | 6915 | 7402 | 1325 | 24605 |
| 156.2425 | 25271 | 7312 | 7799 | 1276 | 18511 |
| 160.7832 | 15304 | 6793 | 7280 | 1118 | 12893 |
| 165.3217 | 10139 | 6803 | 7290 | 1011 | 8709 |
| 169.858 | 7287 | 6957 | 7444 | 1044 | 6019 |
| 174.3921 | 5546 | 7327 | 7814 | 1059 | 4417 |
| 178.9239 | 4596 | 7546 | 8033 | 1010 | 3468 |
| 183.4535 | 3926 | 7062 | 7549 | 1181 | 2961 |
| 187.9808 | 3527 | 5787 | 6274 | 1019 | 2591 |
| 192.5059 | 3352 | 4851 | 5338 | 985 | 2498 |
| 197.0288 | 3110 | 4655 | 5142 | 863 | 2447 |
| 201.5495 | 2795 | 4742 | 5229 | 743 | 2183 |
| 206.0679 | 2274 | 4122 | 4609 | 636 | 1843 |
| 210.5841 | 2010 | 3818 | 4305 | 586 | 1671 |
| 215.0981 | 1801 | 3756 | 4243 | 569 | 1428 |
| 219.6099 | 1640 | 3476 | 3963 | 620 | 1255 |
| 224.1195 | 1540 | 3704 | 4191 | 639 | 1153 |
| 228.6268 | 1448 | 3447 | 3934 | 582 | 1022 |
| 233.1319 | 1340 | 3267 | 3754 | 504 | 963 |
| 237.6348 | 1294 | 3259 | 3746 | 498 | 984 |
| 242.1355 | 1293 | 3091 | 3578 | 461 | 979 |
| 246.634 | 1221 | 2956 | 3443 | 516 | 930 |
| 251.1303 | 1283 | 2916 | 3403 | 495 | 959 |
| 255.6244 | 1274 | 2835 | 3322 | 587 | 962 |
| 260.1163 | 1245 | 2743 | 3230 | 502 | 952 |
| 264.606 | 1230 | 2874 | 3361 | 504 | 925 |
| 269.0934 | 1240 | 2922 | 3409 | 520 | 975 |
| 273.5787 | 1248 | 2661 | 3148 | 526 | 971 |
| 278.0617 | 1258 | 2672 | 3159 | 502 | 913 |
| 282.5426 | 1331 | 2454 | 2941 | 437 | 926 |
| 287.0213 | 1305 | 2525 | 3012 | 499 | 1013 |
| 291.4978 | 1421 | 2827 | 3314 | 511 | 971 |
| 295.9721 | 1403 | 3094 | 3581 | 499 | 1000 |
| 300.4442 | 1452 | 2534 | 3021 | 518 | 1056 |
| 304.9141 | 1457 | 2241 | 2728 | 539 | 1084 |
| 309.3818 | 1527 | 2557 | 3044 | 546 | 1159 |
| 313.8473 | 1501 | 2365 | 2852 | 525 | 1121 |
| 318.3107 | 1583 | 2642 | 3129 | 555 | 1128 |
| 322.7719 | 1545 | 2542 | 3029 | 541 | 1178 |
| 327.2308 | 1546 | 2252 | 2739 | 565 | 1248 |
| 331.6877 | 1614 | 2186 | 2673 | 502 | 1257 |
| 336.1423 | 1693 | 2125 | 2612 | 477 | 1173 |
| 340.5947 | 1631 | 1974 | 2461 | 492 | 1264 |
| 345.045 | 1777 | 1941 | 2428 | 442 | 1308 |
| 349.4931 | 1898 | 2083 | 2570 | 450 | 1301 |
| 353.9391 | 1945 | 1995 | 2482 | 443 | 1348 |
| 358.3828 | 2114 | 1880 | 2367 | 397 | 1511 |
| 362.8244 | 2421 | 2044 | 2531 | 369 | 1590 |
| 367.2638 | 2692 | 2098 | 2585 | 389 | 1814 |
| 371.7011 | 3353 | 2042 | 2529 | 351 | 2056 |
| 376.1362 | 3891 | 1847 | 2334 | 391 | 2258 |
| 380.5691 | 4855 | 1850 | 2337 | 357 | 2647 |
| 384.9999 | 6113 | 1833 | 2320 | 383 | 2903 |
| 389.4285 | 6575 | 1910 | 2397 | 383 | 3177 |
| 393.855 | 7064 | 1847 | 2334 | 354 | 3170 |
| 398.2793 | 6604 | 1765 | 2252 | 379 | 3066 |
| 402.7014 | 5940 | 1738 | 2225 | 322 | 2850 |
| 407.1214 | 4817 | 1874 | 2361 | 342 | 2504 |
| 411.5392 | 3827 | 1693 | 2180 | 355 | 2293 |
| 415.955 | 3140 | 1693 | 2180 | 390 | 1923 |
| 420.3685 | 2626 | 1567 | 2054 | 327 | 1714 |
| 424.7799 | 2297 | 1563 | 2050 | 384 | 1589 |
| 429.1891 | 2143 | 1602 | 2089 | 386 | 1434 |
| 433.5963 | 2023 | 1496 | 1983 | 449 | 1336 |
| 438.0012 | 1899 | 1585 | 2072 | 456 | 1327 |
| 442.404 | 1747 | 1545 | 2032 | 504 | 1257 |
| 446.8047 | 1760 | 1581 | 2068 | 472 | 1280 |
| 451.2033 | 1791 | 1371 | 1858 | 523 | 1292 |
| 455.5997 | 1770 | 1423 | 1910 | 585 | 1223 |
| 459.994 | 1721 | 1393 | 1880 | 618 | 1295 |
| 464.3861 | 1759 | 1487 | 1974 | 655 | 1320 |
| 468.7761 | 1865 | 1597 | 2084 | 690 | 1397 |
| 473.164 | 1943 | 1371 | 1858 | 664 | 1480 |
| 477.5497 | 2058 | 1360 | 1847 | 703 | 1525 |
| 481.9334 | 2227 | 1401 | 1888 | 691 | 1619 |
| 486.3149 | 2420 | 1506 | 1993 | 629 | 1876 |
| 490.6942 | 2925 | 1474 | 1961 | 599 | 2043 |
| 495.0715 | 3565 | 1347 | 1834 | 549 | 2339 |
| 499.4466 | 4272 | 1343 | 1830 | 507 | 2651 |
| 503.8196 | 5393 | 1401 | 1888 | 445 | 2826 |
| 508.1906 | 6250 | 1437 | 1924 | 440 | 3066 |
| 512.5593 | 6812 | 1372 | 1859 | 465 | 3167 |
| 516.926 | 6815 | 1251 | 1738 | 413 | 2954 |
| 521.2905 | 6248 | 1153 | 1640 | 440 | 2868 |
| 525.653 | 5421 | 1150 | 1637 | 448 | 2580 |
| 530.0133 | 4773 | 1268 | 1755 | 438 | 2416 |
| 534.3715 | 4081 | 1219 | 1706 | 448 | 2089 |
| 538.7277 | 3554 | 1161 | 1648 | 482 | 1988 |
| 543.0817 | 3170 | 1100 | 1587 | 443 | 1897 |
| 547.4336 | 2791 | 1259 | 1746 | 476 | 1893 |
| 551.7834 | 2755 | 1220 | 1707 | 504 | 1772 |
| 556.1311 | 2588 | 1140 | 1627 | 502 | 1684 |
| 560.4767 | 2417 | 1021 | 1508 | 479 | 1637 |
| 564.8202 | 2407 | 1011 | 1498 | 482 | 1660 |
| 569.1616 | 2453 | 1062 | 1549 | 475 | 1679 |
| 573.5009 | 2369 | 1213 | 1700 | 462 | 1641 |
| 577.8381 | 2387 | 1103 | 1590 | 459 | 1622 |
| 582.1732 | 2559 | 1150 | 1637 | 516 | 1784 |
| 586.5063 | 2608 | 1237 | 1724 | 455 | 1838 |
| 590.8372 | 2764 | 1000 | 1487 | 448 | 1886 |
| 595.1661 | 3062 | 1028 | 1515 | 393 | 2038 |
| 599.4929 | 3338 | 1068 | 1555 | 397 | 2330 |
| 603.8175 | 3800 | 1073 | 1560 | 415 | 2473 |
| 608.1401 | 4529 | 1040 | 1527 | 421 | 2829 |
| 612.4606 | 5272 | 1013 | 1500 | 429 | 3272 |
| 616.7791 | 6669 | 923 | 1410 | 510 | 3756 |
| 621.0955 | 7836 | 1063 | 1550 | 507 | 4244 |
| 625.4097 | 9680 | 1055 | 1542 | 521 | 4913 |
| 629.722 | 11519 | 1081 | 1568 | 511 | 5013 |
| 634.0321 | 12558 | 1081 | 1568 | 548 | 5311 |
| 638.3401 | 13044 | 1088 | 1575 | 622 | 5072 |
| 642.6461 | 11959 | 1057 | 1544 | 597 | 4644 |
| 646.9501 | 10399 | 1009 | 1496 | 659 | 4150 |
| 651.2519 | 8640 | 1056 | 1543 | 679 | 3658 |
| 655.5517 | 6890 | 967 | 1454 | 687 | 3229 |
| 659.8494 | 5365 | 1031 | 1518 | 640 | 2721 |
| 664.1451 | 4513 | 1015 | 1502 | 747 | 2410 |
| 668.4387 | 3770 | 915 | 1402 | 758 | 2181 |
| 672.7302 | 3319 | 925 | 1412 | 769 | 1946 |
| 677.0197 | 2854 | 1012 | 1499 | 845 | 1776 |
| 681.3071 | 2614 | 961 | 1448 | 867 | 1581 |
| 685.5925 | 2313 | 989 | 1476 | 935 | 1529 |
| 689.8757 | 2094 | 1001 | 1488 | 932 | 1477 |
| 694.157 | 1888 | 1083 | 1570 | 875 | 1394 |
| 698.4362 | 1769 | 989 | 1476 | 900 | 1323 |
| 702.7133 | 1611 | 948 | 1435 | 801 | 1165 |
| 706.9884 | 1496 | 863 | 1350 | 719 | 1068 |
| 711.2615 | 1406 | 917 | 1404 | 629 | 1099 |
| 715.5325 | 1376 | 933 | 1420 | 601 | 1042 |
| 719.8015 | 1272 | 899 | 1386 | 504 | 962 |
| 724.0684 | 1205 | 900 | 1387 | 438 | 912 |
| 728.3332 | 1142 | 893 | 1380 | 455 | 904 |
| 732.596 | 1105 | 933 | 1420 | 372 | 875 |
| 736.8568 | 1059 | 922 | 1409 | 355 | 879 |
| 741.1156 | 1024 | 1023 | 1510 | 368 | 830 |
| 745.3723 | 985 | 915 | 1402 | 355 | 781 |
| 749.627 | 960 | 938 | 1425 | 331 | 814 |
| 753.8796 | 1011 | 929 | 1416 | 311 | 803 |
| 758.1302 | 933 | 836 | 1323 | 301 | 789 |
| 762.3788 | 974 | 878 | 1365 | 311 | 743 |
| 766.6254 | 950 | 904 | 1391 | 260 | 793 |
| 770.8699 | 1061 | 836 | 1323 | 266 | 801 |
| 775.1124 | 998 | 886 | 1373 | 263 | 797 |
| 779.3528 | 1005 | 895 | 1382 | 234 | 767 |
| 783.5913 | 978 | 957 | 1444 | 276 | 766 |
| 787.8278 | 1041 | 919 | 1406 | 243 | 732 |
| 792.0621 | 1078 | 913 | 1400 | 248 | 775 |
| 796.2946 | 1019 | 883 | 1370 | 237 | 767 |
| 800.5249 | 1053 | 930 | 1417 | 202 | 802 |
| 804.7532 | 1026 | 986 | 1473 | 199 | 746 |
| 808.9796 | 938 | 942 | 1429 | 205 | 792 |
| 813.2039 | 1004 | 882 | 1369 | 184 | 720 |
| 817.4262 | 907 | 911 | 1398 | 208 | 699 |
| 821.6465 | 896 | 950 | 1437 | 213 | 673 |
| 825.8648 | 880 | 972 | 1459 | 215 | 668 |
| 830.0811 | 828 | 954 | 1441 | 213 | 746 |
| 834.2954 | 850 | 979 | 1466 | 225 | 671 |
| 838.5077 | 797 | 917 | 1404 | 212 | 644 |
| 842.718 | 794 | 935 | 1422 | 205 | 678 |
| 846.9262 | 805 | 934 | 1421 | 187 | 631 |
| 851.1324 | 740 | 940 | 1427 | 183 | 624 |
| 855.3367 | 761 | 884 | 1371 | 191 | 577 |
| 859.539 | 750 | 911 | 1398 | 215 | 606 |
| 863.7393 | 750 | 924 | 1411 | 205 | 627 |
| 867.9376 | 689 | 936 | 1423 | 222 | 570 |
| 872.1339 | 673 | 889 | 1376 | 208 | 606 |
| 876.3281 | 714 | 978 | 1465 | 202 | 572 |
| 880.5204 | 678 | 841 | 1328 | 215 | 607 |
| 884.7108 | 621 | 855 | 1342 | 227 | 555 |
| 888.899 | 600 | 944 | 1431 | 176 | 542 |
| 893.0854 | 619 | 948 | 1435 | 182 | 556 |
| 897.2697 | 579 | 871 | 1358 | 193 | 545 |
| 901.4521 | 575 | 891 | 1378 | 217 | 544 |
| 905.6325 | 577 | 870 | 1357 | 207 | 571 |
| 909.8109 | 586 | 954 | 1441 | 200 | 531 |
| 913.9873 | 624 | 932 | 1419 | 213 | 512 |
| 918.1617 | 567 | 989 | 1476 | 204 | 490 |
| 922.3342 | 529 | 1046 | 1533 | 211 | 497 |
| 926.5047 | 556 | 933 | 1420 | 208 | 502 |
| 930.6732 | 557 | 916 | 1403 | 197 | 513 |
| 934.8398 | 502 | 969 | 1456 | 224 | 507 |
| 939.0043 | 543 | 959 | 1446 | 185 | 510 |
| 943.1669 | 450 | 959 | 1446 | 166 | 489 |
| 947.3276 | 476 | 1037 | 1524 | 188 | 479 |
| 951.4862 | 481 | 1071 | 1558 | 204 | 482 |
| 955.6429 | 498 | 1035 | 1522 | 191 | 449 |
| 959.7977 | 439 | 1120 | 1607 | 206 | 442 |
| 963.9504 | 464 | 1103 | 1590 | 209 | 460 |
| 968.1013 | 444 | 1142 | 1629 | 178 | 486 |
| 972.2501 | 418 | 1011 | 1498 | 206 | 453 |
| 976.397 | 407 | 1094 | 1581 | 199 | 459 |
| 980.5419 | 433 | 1076 | 1563 | 180 | 434 |
| 984.6849 | 373 | 1109 | 1596 | 179 | 432 |
| 988.8259 | 391 | 1150 | 1637 | 206 | 458 |
| 992.965 | 404 | 1130 | 1617 | 182 | 421 |
| 997.1021 | 371 | 1107 | 1594 | 191 | 417 |
| 1001.237 | 389 | 1114 | 1601 | 197 | 423 |
| 1005.37 | 375 | 1141 | 1628 | 202 | 415 |
| 1009.502 | 370 | 1204 | 1691 | 163 | 418 |
| 1013.631 | 371 | 1203 | 1690 | 164 | 429 |
| 1017.758 | 371 | 1336 | 1823 | 203 | 407 |
| 1021.884 | 351 | 1213 | 1700 | 215 | 402 |
| 1026.007 | 385 | 1206 | 1693 | 203 | 392 |
| 1030.129 | 404 | 1262 | 1749 | 197 | 397 |
| 1034.248 | 347 | 1190 | 1677 | 203 | 430 |
| 1038.366 | 361 | 1289 | 1776 | 196 | 419 |
| 1042.482 | 308 | 1257 | 1744 | 184 | 384 |
| 1046.596 | 349 | 1333 | 1820 | 207 | 405 |
| 1050.707 | 352 | 1259 | 1746 | 174 | 409 |
| 1054.817 | 355 | 1294 | 1781 | 186 | 404 |
| 1058.925 | 321 | 1371 | 1858 | 205 | 391 |
| 1063.031 | 323 | 1371 | 1858 | 180 | 398 |
| 1067.135 | 319 | 1394 | 1881 | 195 | 358 |
| 1071.238 | 342 | 1391 | 1878 | 191 | 414 |
| 1075.338 | 331 | 1428 | 1915 | 208 | 376 |
| 1079.436 | 335 | 1437 | 1924 | 216 | 418 |
| 1083.533 | 321 | 1497 | 1984 | 220 | 372 |
| 1087.627 | 303 | 1555 | 2042 | 210 | 371 |
| 1091.72 | 329 | 1619 | 2106 | 245 | 428 |
| 1095.811 | 300 | 1534 | 2021 | 226 | 364 |
| 1099.899 | 304 | 1651 | 2138 | 217 | 335 |
| 1103.986 | 305 | 1660 | 2147 | 206 | 409 |
| 1108.071 | 321 | 1625 | 2112 | 221 | 365 |
| 1112.154 | 326 | 1651 | 2138 | 202 | 407 |
| 1116.235 | 292 | 1697 | 2184 | 225 | 393 |
| 1120.314 | 326 | 1660 | 2147 | 210 | 358 |
| 1124.391 | 314 | 1642 | 2129 | 243 | 404 |
| 1128.467 | 329 | 1740 | 2227 | 208 | 383 |
| 1132.54 | 290 | 1839 | 2326 | 188 | 435 |
| 1136.612 | 293 | 1872 | 2359 | 209 | 398 |
| 1140.681 | 315 | 1801 | 2288 | 207 | 364 |
| 1144.749 | 266 | 1895 | 2382 | 163 | 372 |
| 1148.815 | 306 | 1901 | 2388 | 195 | 382 |
| 1152.879 | 290 | 1916 | 2403 | 228 | 364 |
| 1156.941 | 262 | 2017 | 2504 | 184 | 389 |
| 1161.001 | 289 | 2031 | 2518 | 228 | 390 |
| 1165.059 | 291 | 2048 | 2535 | 186 | 389 |
| 1169.115 | 264 | 2059 | 2546 | 192 | 393 |
| 1173.17 | 314 | 2210 | 2697 | 215 | 378 |
| 1177.222 | 299 | 2114 | 2601 | 199 | 373 |
| 1181.273 | 300 | 2227 | 2714 | 214 | 415 |
| 1185.322 | 298 | 2338 | 2825 | 171 | 372 |
| 1189.369 | 274 | 2352 | 2839 | 211 | 409 |
| 1193.414 | 279 | 2421 | 2908 | 187 | 418 |
| 1197.457 | 327 | 2399 | 2886 | 215 | 383 |
| 1201.498 | 275 | 2487 | 2974 | 180 | 391 |
| 1205.537 | 276 | 2611 | 3098 | 200 | 397 |
| 1209.575 | 300 | 2824 | 3311 | 201 | 436 |
| 1213.61 | 289 | 2901 | 3388 | 199 | 400 |
| 1217.644 | 310 | 2915 | 3402 | 182 | 368 |
| 1221.676 | 315 | 3164 | 3651 | 201 | 410 |
| 1225.706 | 277 | 3142 | 3629 | 201 | 381 |
| 1229.734 | 309 | 3352 | 3839 | 176 | 399 |
| 1233.76 | 285 | 3427 | 3914 | 179 | 399 |
| 1237.784 | 320 | 3540 | 4027 | 195 | 405 |
| 1241.807 | 269 | 3725 | 4212 | 198 | 438 |
| 1245.827 | 342 | 3981 | 4468 | 222 | 421 |
| 1249.846 | 293 | 4174 | 4661 | 214 | 410 |
| 1253.862 | 300 | 4308 | 4795 | 206 | 405 |
| 1257.877 | 308 | 4560 | 5047 | 222 | 408 |
| 1261.891 | 312 | 4827 | 5314 | 208 | 413 |
| 1265.902 | 295 | 4836 | 5323 | 231 | 433 |
| 1269.911 | 332 | 5303 | 5790 | 241 | 430 |
| 1273.918 | 269 | 5606 | 6093 | 246 | 411 |
| 1277.924 | 326 | 5904 | 6391 | 237 | 412 |
| 1281.928 | 293 | 6285 | 6772 | 258 | 431 |
| 1285.93 | 300 | 6612 | 7099 | 249 | 439 |
| 1289.93 | 280 | 7007 | 7494 | 259 | 419 |
| 1293.928 | 308 | 7066 | 7553 | 234 | 455 |
| 1297.924 | 332 | 7704 | 8191 | 235 | 437 |
| 1301.919 | 306 | 8229 | 8716 | 257 | 465 |
| 1305.911 | 270 | 8642 | 9529 | 260 | 431 |
| 1309.902 | 283 | 9091 | 9978 | 251 | 433 |
| 1313.891 | 280 | 9684 | 10771 | 240 | 432 |
| 1317.878 | 301 | 10043 | 11530 | 218 | 441 |
| 1321.863 | 302 | 10397 | 11884 | 270 | 455 |
| 1325.847 | 292 | 10917 | 12104 | 238 | 486 |
| 1329.828 | 289 | 11213 | 12700 | 227 | 435 |
| 1333.808 | 281 | 11637 | 13124 | 240 | 455 |
| 1337.786 | 308 | 11960 | 13743 | 251 | 395 |
| 1341.762 | 317 | 12177 | 14254 | 230 | 471 |
| 1345.736 | 335 | 12448 | 14868 | 252 | 426 |
| 1349.708 | 295 | 12135 | 14855 | 276 | 435 |
| 1353.679 | 303 | 11736 | 14223 | 220 | 441 |
| 1357.647 | 299 | 11073 | 13769 | 210 | 431 |
| 1361.614 | 344 | 10870 | 13527 | 226 | 431 |
| 1365.579 | 321 | 10399 | 12886 | 207 | 447 |
| 1369.542 | 295 | 10101 | 10588 | 199 | 451 |
| 1373.504 | 286 | 9392 | 9879 | 203 | 427 |
| 1377.463 | 294 | 8855 | 9342 | 208 | 399 |
| 1381.421 | 325 | 8596 | 9083 | 188 | 472 |
| 1385.377 | 299 | 8422 | 8909 | 224 | 455 |
| 1389.331 | 305 | 7786 | 8273 | 197 | 430 |
| 1393.283 | 314 | 7627 | 8114 | 223 | 431 |
| 1397.233 | 297 | 7274 | 7761 | 218 | 423 |
| 1401.182 | 332 | 6608 | 7095 | 185 | 452 |
| 1405.128 | 288 | 6506 | 6993 | 173 | 419 |
| 1409.073 | 317 | 6181 | 6668 | 182 | 439 |
| 1413.016 | 311 | 6117 | 6604 | 194 | 403 |
| 1416.958 | 286 | 5810 | 6297 | 180 | 434 |
| 1420.897 | 264 | 5477 | 5964 | 149 | 434 |
| 1424.835 | 308 | 5519 | 6006 | 178 | 401 |
| 1428.77 | 316 | 5162 | 5649 | 160 | 415 |
| 1432.704 | 314 | 4989 | 5476 | 144 | 413 |
| 1436.637 | 325 | 5190 | 5677 | 157 | 431 |
| 1440.567 | 299 | 5004 | 5491 | 175 | 395 |
| 1444.496 | 292 | 4885 | 5372 | 175 | 413 |
| 1448.422 | 295 | 5022 | 5509 | 161 | 401 |
| 1452.347 | 302 | 4958 | 5445 | 162 | 462 |
| 1456.27 | 301 | 4688 | 5175 | 155 | 391 |
| 1460.192 | 309 | 4890 | 5377 | 171 | 418 |
| 1464.111 | 281 | 4836 | 5323 | 162 | 386 |
| 1468.029 | 283 | 4893 | 5380 | 157 | 389 |
| 1471.945 | 319 | 4911 | 5398 | 154 | 418 |
| 1475.859 | 267 | 4832 | 5319 | 155 | 430 |
| 1479.771 | 304 | 4720 | 5207 | 116 | 401 |
| 1483.682 | 303 | 4840 | 5327 | 159 | 418 |
| 1487.591 | 307 | 4960 | 5447 | 150 | 441 |
| 1491.498 | 282 | 4975 | 5462 | 161 | 457 |
| 1495.403 | 269 | 5039 | 5526 | 140 | 425 |
| 1499.306 | 271 | 5011 | 5498 | 167 | 426 |
| 1503.208 | 313 | 5303 | 5790 | 120 | 422 |
| 1507.108 | 319 | 5576 | 6063 | 147 | 447 |
| 1511.006 | 281 | 5602 | 6089 | 145 | 433 |
| 1514.902 | 298 | 5895 | 6382 | 145 | 432 |
| 1518.796 | 268 | 5882 | 6369 | 162 | 460 |
| 1522.689 | 302 | 6170 | 6657 | 117 | 424 |
| 1526.58 | 305 | 6447 | 6934 | 137 | 457 |
| 1530.469 | 296 | 7037 | 7524 | 142 | 489 |
| 1534.356 | 264 | 7358 | 7845 | 124 | 440 |
| 1538.242 | 312 | 7531 | 8018 | 119 | 427 |
| 1542.125 | 285 | 8210 | 8697 | 125 | 463 |
| 1546.007 | 305 | 8626 | 9113 | 116 | 469 |
| 1549.888 | 261 | 9379 | 9866 | 162 | 471 |
| 1553.766 | 313 | 10275 | 10762 | 144 | 429 |
| 1557.643 | 314 | 10610 | 11097 | 112 | 454 |
| 1561.518 | 320 | 11727 | 12214 | 144 | 481 |
| 1565.391 | 347 | 12247 | 12734 | 126 | 481 |
| 1569.262 | 317 | 13280 | 13767 | 123 | 411 |
| 1573.131 | 319 | 13233 | 13720 | 94 | 470 |
| 1576.999 | 276 | 13225 | 13712 | 122 | 482 |
| 1580.865 | 321 | 12686 | 13173 | 104 | 496 |
| 1584.73 | 338 | 12283 | 12770 | 123 | 464 |
| 1588.592 | 314 | 11582 | 12069 | 128 | 506 |
| 1592.453 | 331 | 10743 | 11230 | 134 | 490 |
| 1596.312 | 323 | 10466 | 10953 | 140 | 469 |
| 1600.169 | 313 | 9520 | 10007 | 104 | 519 |
| 1604.024 | 300 | 8847 | 9334 | 111 | 457 |
| 1607.878 | 330 | 8170 | 8657 | 123 | 479 |
| 1611.73 | 326 | 7578 | 8065 | 148 | 517 |
| 1615.58 | 366 | 6574 | 7061 | 115 | 459 |
| 1619.429 | 285 | 5659 | 6146 | 115 | 476 |
| 1623.275 | 322 | 4811 | 5298 | 126 | 468 |
| 1627.12 | 346 | 4096 | 4583 | 133 | 475 |
| 1630.964 | 339 | 3626 | 4113 | 93 | 481 |
| 1634.805 | 318 | 3076 | 3563 | 133 | 470 |
| 1638.645 | 322 | 2616 | 3103 | 156 | 434 |
| 1642.482 | 333 | 2297 | 2784 | 137 | 428 |
| 1646.319 | 321 | 1962 | 2449 | 140 | 431 |
| 1650.153 | 291 | 1813 | 2300 | 126 | 447 |
| 1653.986 | 348 | 1682 | 2169 | 140 | 466 |
| 1657.817 | 311 | 1503 | 1990 | 121 | 453 |
| 1661.646 | 287 | 1467 | 1954 | 126 | 453 |
| 1665.473 | 297 | 1366 | 1853 | 124 | 424 |
| 1669.299 | 283 | 1301 | 1788 | 115 | 416 |
| 1673.123 | 312 | 1251 | 1738 | 113 | 408 |
| 1676.945 | 344 | 1214 | 1701 | 128 | 443 |
| 1680.766 | 282 | 1225 | 1712 | 136 | 467 |
| 1684.585 | 302 | 1171 | 1658 | 127 | 438 |
| 1688.402 | 300 | 1185 | 1672 | 117 | 440 |
| 1692.217 | 341 | 1159 | 1646 | 137 | 423 |
| 1696.031 | 348 | 1090 | 1577 | 120 | 433 |
| 1699.842 | 344 | 1055 | 1542 | 112 | 411 |
| 1703.653 | 304 | 958 | 1445 | 144 | 404 |
| 1707.461 | 304 | 1038 | 1525 | 116 | 398 |
| 1711.268 | 322 | 1068 | 1555 | 117 | 394 |
| 1715.073 | 319 | 1037 | 1524 | 117 | 435 |
| 1718.876 | 298 | 999 | 1486 | 118 | 426 |
| 1722.677 | 313 | 1060 | 1547 | 117 | 439 |
| 1726.477 | 315 | 1020 | 1507 | 136 | 403 |
| 1730.275 | 329 | 1003 | 1490 | 109 | 452 |
| 1734.071 | 294 | 1053 | 1540 | 133 | 403 |
| 1737.866 | 251 | 1004 | 1491 | 100 | 424 |
| 1741.659 | 268 | 1025 | 1512 | 96 | 388 |
| 1745.45 | 249 | 982 | 1469 | 110 | 428 |
| 1749.24 | 290 | 878 | 1365 | 122 | 437 |
| 1753.027 | 305 | 962 | 1449 | 126 | 416 |
| 1756.813 | 272 | 1018 | 1505 | 101 | 380 |
| 1760.598 | 270 | 885 | 1372 | 101 | 422 |
| 1764.38 | 290 | 882 | 1369 | 123 | 411 |
| 1768.161 | 301 | 907 | 1394 | 130 | 432 |
| 1771.94 | 247 | 872 | 1359 | 110 | 412 |
| 1775.718 | 306 | 819 | 1306 | 88 | 389 |
| 1779.494 | 268 | 865 | 1352 | 122 | 422 |
| 1783.268 | 275 | 882 | 1369 | 122 | 434 |
| 1787.04 | 289 | 838 | 1325 | 126 | 405 |
| 1790.811 | 280 | 810 | 1297 | 134 | 429 |
| 1794.58 | 254 | 762 | 1249 | 122 | 386 |
| 1798.347 | 281 | 751 | 1238 | 116 | 415 |
| 1802.113 | 267 | 795 | 1282 | 122 | 411 |
| 1805.876 | 270 | 746 | 1233 | 99 | 402 |
| 1809.639 | 246 | 713 | 1200 | 123 | 391 |
| 1813.399 | 255 | 637 | 1124 | 110 | 387 |
| 1817.158 | 265 | 728 | 1215 | 122 | 446 |
| 1820.915 | 265 | 722 | 1209 | 120 | 406 |
| 1824.67 | 265 | 679 | 1166 | 106 | 399 |
| 1828.424 | 271 | 763 | 1250 | 87 | 380 |
| 1832.176 | 295 | 759 | 1246 | 108 | 398 |
| 1835.927 | 280 | 725 | 1212 | 107 | 398 |
| 1839.675 | 262 | 721 | 1208 | 127 | 397 |
| 1843.422 | 279 | 654 | 1141 | 124 | 366 |
| 1847.167 | 241 | 695 | 1182 | 112 | 370 |
| 1850.911 | 262 | 651 | 1138 | 108 | 431 |
| 1854.653 | 263 | 640 | 1127 | 116 | 413 |
| 1858.393 | 277 | 654 | 1141 | 110 | 393 |
| 1862.132 | 290 | 674 | 1161 | 98 | 432 |
| 1865.869 | 267 | 641 | 1128 | 82 | 386 |
| 1869.604 | 265 | 604 | 1091 | 88 | 428 |
| 1873.337 | 248 | 625 | 1112 | 120 | 382 |
| 1877.069 | 286 | 616 | 1103 | 120 | 431 |
| 1880.799 | 274 | 528 | 1015 | 105 | 421 |
| 1884.528 | 244 | 646 | 1133 | 92 | 430 |
| 1888.255 | 273 | 588 | 1075 | 90 | 394 |
| 1891.98 | 284 | 546 | 1033 | 109 | 394 |
| 1895.703 | 283 | 560 | 1047 | 82 | 401 |
| 1899.425 | 236 | 560 | 1047 | 97 | 407 |
| 1903.145 | 239 | 613 | 1100 | 100 | 395 |
| 1906.863 | 251 | 592 | 1079 | 97 | 416 |
| 1910.58 | 273 | 610 | 1097 | 107 | 392 |
| 1914.295 | 254 | 544 | 1031 | 97 | 394 |
| 1918.009 | 290 | 630 | 1117 | 96 | 423 |
| 1921.721 | 258 | 601 | 1088 | 104 | 417 |
| 1925.431 | 250 | 524 | 1011 | 99 | 403 |
| 1929.139 | 230 | 588 | 1075 | 89 | 426 |
| 1932.846 | 267 | 560 | 1047 | 105 | 406 |
| 1936.551 | 257 | 514 | 1001 | 120 | 437 |
| 1940.255 | 252 | 601 | 1088 | 87 | 410 |
| 1943.957 | 250 | 550 | 1037 | 100 | 406 |
| 1947.657 | 268 | 569 | 1056 | 98 | 335 |
| 1951.355 | 288 | 573 | 1060 | 91 | 407 |
| 1955.052 | 257 | 538 | 1025 | 104 | 376 |
| 1958.747 | 288 | 539 | 1026 | 119 | 406 |
| 1962.441 | 265 | 526 | 1013 | 101 | 423 |
| 1966.133 | 266 | 538 | 1025 | 102 | 399 |
| 1969.823 | 243 | 509 | 996 | 103 | 382 |
| 1973.512 | 247 | 453 | 940 | 103 | 433 |
| 1977.199 | 274 | 570 | 1057 | 111 | 404 |
| 1980.884 | 270 | 508 | 995 | 86 | 448 |
| 1984.568 | 283 | 519 | 1006 | 96 | 407 |
| 1988.25 | 250 | 547 | 1034 | 117 | 385 |
| 1991.93 | 258 | 489 | 976 | 115 | 392 |
| 1995.609 | 282 | 467 | 954 | 89 | 415 |
| 1999.286 | 272 | 529 | 1016 | 72 | 402 |
| 2002.961 | 249 | 485 | 972 | 112 | 370 |
| 2006.635 | 276 | 563 | 1050 | 92 | 387 |
| 2010.307 | 254 | 513 | 1000 | 91 | 381 |
| 2013.978 | 288 | 517 | 1004 | 104 | 377 |
| 2017.647 | 270 | 530 | 1017 | 104 | 387 |
| 2021.314 | 266 | 589 | 1076 | 97 | 416 |
| 2024.98 | 244 | 507 | 994 | 91 | 397 |
| 2028.644 | 254 | 490 | 977 | 80 | 384 |
| 2032.306 | 263 | 571 | 1058 | 87 | 414 |
| 2035.967 | 269 | 486 | 973 | 112 | 415 |
| 2039.626 | 260 | 515 | 1002 | 104 | 403 |
| 2043.284 | 260 | 498 | 985 | 89 | 436 |
| 2046.94 | 272 | 509 | 996 | 96 | 348 |
| 2050.594 | 271 | 462 | 949 | 118 | 404 |
| 2054.247 | 241 | 440 | 927 | 110 | 430 |
| 2057.897 | 271 | 478 | 965 | 98 | 408 |
| 2061.547 | 286 | 501 | 988 | 85 | 389 |
| 2065.195 | 269 | 515 | 1002 | 73 | 425 |
| 2068.841 | 257 | 451 | 938 | 111 | 367 |
| 2072.485 | 283 | 467 | 954 | 99 | 429 |
| 2076.128 | 240 | 492 | 979 | 73 | 418 |
| 2079.77 | 248 | 449 | 936 | 89 | 423 |
| 2083.409 | 279 | 491 | 978 | 87 | 408 |
| 2087.047 | 286 | 455 | 942 | 85 | 414 |
| 2090.684 | 234 | 481 | 968 | 66 | 369 |
| 2094.319 | 281 | 486 | 973 | 93 | 368 |
| 2097.952 | 290 | 439 | 926 | 69 | 394 |
| 2101.583 | 282 | 483 | 970 | 98 | 416 |
| 2105.213 | 286 | 510 | 997 | 87 | 401 |
| 2108.842 | 271 | 527 | 1014 | 92 | 427 |
| 2112.469 | 259 | 455 | 942 | 88 | 392 |
| 2116.094 | 267 | 420 | 907 | 92 | 370 |
| 2119.718 | 250 | 487 | 974 | 104 | 432 |
| 2123.339 | 233 | 457 | 944 | 88 | 399 |
| 2126.96 | 283 | 447 | 934 | 97 | 395 |
| 2130.579 | 265 | 399 | 886 | 100 | 396 |
| 2134.196 | 269 | 498 | 985 | 81 | 405 |
| 2137.811 | 265 | 454 | 941 | 94 | 383 |
| 2141.425 | 285 | 392 | 879 | 82 | 424 |
| 2145.038 | 258 | 418 | 905 | 84 | 392 |
| 2148.648 | 244 | 404 | 891 | 90 | 395 |
| 2152.258 | 240 | 408 | 895 | 95 | 411 |
| 2155.865 | 273 | 482 | 969 | 65 | 428 |
| 2159.471 | 256 | 421 | 908 | 90 | 413 |
| 2163.075 | 275 | 400 | 887 | 65 | 392 |
| 2166.678 | 255 | 455 | 942 | 93 | 368 |
| 2170.28 | 276 | 462 | 949 | 88 | 358 |
| 2173.879 | 264 | 468 | 955 | 77 | 426 |
| 2177.477 | 280 | 421 | 908 | 72 | 400 |
| 2181.073 | 258 | 416 | 903 | 83 | 408 |
| 2184.668 | 270 | 416 | 903 | 90 | 399 |
| 2188.262 | 256 | 398 | 885 | 77 | 421 |
| 2191.854 | 221 | 434 | 921 | 98 | 407 |
| 2195.444 | 257 | 370 | 857 | 98 | 412 |
| 2199.032 | 262 | 415 | 902 | 51 | 422 |
| 2202.619 | 287 | 449 | 936 | 94 | 408 |
| 2206.204 | 274 | 443 | 930 | 78 | 407 |
| 2209.788 | 266 | 426 | 913 | 99 | 398 |
| 2213.37 | 274 | 460 | 947 | 81 | 394 |
| 2216.951 | 257 | 445 | 932 | 71 | 401 |
| 2220.53 | 236 | 405 | 892 | 72 | 404 |
| 2224.107 | 260 | 412 | 899 | 89 | 403 |
| 2227.683 | 268 | 373 | 860 | 83 | 400 |
| 2231.258 | 261 | 427 | 914 | 75 | 391 |
| 2234.83 | 275 | 384 | 871 | 82 | 417 |
| 2238.402 | 248 | 392 | 879 | 107 | 412 |
| 2241.971 | 274 | 385 | 872 | 74 | 397 |
| 2245.539 | 250 | 423 | 910 | 75 | 406 |
| 2249.106 | 255 | 461 | 948 | 72 | 422 |
| 2252.671 | 271 | 362 | 849 | 94 | 441 |
| 2256.234 | 237 | 380 | 867 | 79 | 388 |
| 2259.796 | 291 | 420 | 907 | 86 | 425 |
| 2263.356 | 287 | 455 | 942 | 88 | 400 |
| 2266.915 | 257 | 417 | 904 | 77 | 402 |
| 2270.472 | 284 | 432 | 919 | 83 | 405 |
| 2274.027 | 266 | 435 | 922 | 77 | 395 |
| 2277.581 | 251 | 436 | 923 | 87 | 412 |
| 2281.134 | 262 | 346 | 833 | 76 | 430 |
| 2284.684 | 255 | 433 | 920 | 77 | 365 |
| 2288.234 | 239 | 433 | 920 | 68 | 420 |
| 2291.781 | 282 | 414 | 901 | 94 | 410 |
| 2295.328 | 260 | 423 | 910 | 62 | 385 |
| 2298.872 | 271 | 471 | 958 | 78 | 386 |
| 2302.415 | 273 | 422 | 909 | 72 | 457 |
| 2305.957 | 245 | 396 | 883 | 85 | 355 |
| 2309.497 | 267 | 370 | 857 | 83 | 395 |
| 2313.035 | 276 | 412 | 899 | 82 | 372 |
| 2316.572 | 253 | 455 | 942 | 75 | 400 |
| 2320.107 | 270 | 500 | 987 | 71 | 378 |
| 2323.641 | 269 | 420 | 907 | 71 | 453 |
| 2327.173 | 284 | 486 | 973 | 75 | 414 |
| 2330.704 | 226 | 448 | 935 | 81 | 423 |
| 2334.233 | 249 | 435 | 922 | 72 | 386 |
| 2337.761 | 253 | 444 | 931 | 81 | 399 |
| 2341.287 | 232 | 360 | 847 | 82 | 415 |
| 2344.811 | 263 | 465 | 952 | 74 | 464 |
| 2348.334 | 274 | 425 | 912 | 72 | 409 |
| 2351.856 | 258 | 416 | 903 | 84 | 389 |
| 2355.376 | 281 | 426 | 913 | 75 | 405 |
| 2358.894 | 256 | 409 | 896 | 64 | 379 |
| 2362.411 | 276 | 475 | 962 | 100 | 429 |
| 2365.926 | 256 | 396 | 883 | 93 | 403 |
| 2369.44 | 262 | 395 | 882 | 76 | 408 |
| 2372.952 | 273 | 408 | 895 | 78 | 372 |
| 2376.463 | 243 | 431 | 918 | 76 | 373 |
| 2379.972 | 263 | 467 | 954 | 77 | 410 |
| 2383.479 | 258 | 418 | 905 | 80 | 364 |
| 2386.986 | 262 | 472 | 959 | 86 | 412 |
| 2390.49 | 214 | 496 | 983 | 78 | 403 |
| 2393.993 | 243 | 448 | 935 | 75 | 420 |
| 2397.495 | 268 | 516 | 1003 | 69 | 391 |
| 2400.995 | 266 | 540 | 1027 | 78 | 403 |
| 2404.493 | 271 | 518 | 1005 | 90 | 430 |
| 2407.99 | 251 | 498 | 985 | 78 | 398 |
| 2411.486 | 261 | 532 | 1019 | 81 | 403 |
| 2414.979 | 281 | 484 | 971 | 87 | 384 |
| 2418.472 | 256 | 562 | 1049 | 69 | 416 |
| 2421.963 | 280 | 496 | 983 | 67 | 390 |
| 2425.452 | 249 | 537 | 1024 | 95 | 409 |
| 2428.94 | 261 | 543 | 1030 | 87 | 390 |
| 2432.426 | 246 | 638 | 1125 | 69 | 371 |
| 2435.911 | 250 | 619 | 1106 | 79 | 411 |
| 2439.394 | 249 | 582 | 1069 | 88 | 414 |
| 2442.876 | 247 | 597 | 1084 | 95 | 396 |
| 2446.356 | 273 | 567 | 1054 | 78 | 389 |
| 2449.835 | 244 | 590 | 1077 | 83 | 429 |
| 2453.312 | 275 | 630 | 1117 | 66 | 356 |
| 2456.788 | 242 | 594 | 1081 | 75 | 434 |
| 2460.262 | 264 | 650 | 1137 | 75 | 406 |
| 2463.735 | 247 | 599 | 1086 | 82 | 404 |
| 2467.206 | 267 | 671 | 1158 | 96 | 403 |
| 2470.676 | 282 | 622 | 1109 | 74 | 454 |
| 2474.144 | 250 | 602 | 1089 | 94 | 394 |
| 2477.611 | 261 | 582 | 1069 | 95 | 410 |
| 2481.076 | 290 | 679 | 1166 | 82 | 386 |
| 2484.539 | 240 | 684 | 1171 | 55 | 413 |
| 2488.001 | 247 | 670 | 1157 | 81 | 410 |
| 2491.462 | 258 | 658 | 1145 | 75 | 401 |
| 2494.921 | 263 | 587 | 1074 | 68 | 399 |
| 2498.379 | 243 | 649 | 1136 | 65 | 385 |
| 2501.835 | 299 | 634 | 1121 | 77 | 392 |
| 2505.29 | 268 | 653 | 1140 | 61 | 405 |
| 2508.743 | 244 | 620 | 1107 | 66 | 404 |
| 2512.195 | 248 | 605 | 1092 | 75 | 433 |
| 2515.645 | 234 | 624 | 1111 | 74 | 395 |
| 2519.094 | 261 | 615 | 1102 | 73 | 395 |
| 2522.541 | 256 | 696 | 1183 | 75 | 397 |
| 2525.987 | 248 | 733 | 1220 | 55 | 411 |
| 2529.431 | 224 | 668 | 1155 | 65 | 392 |
| 2532.874 | 285 | 643 | 1130 | 67 | 411 |
| 2536.315 | 283 | 659 | 1146 | 71 | 429 |
| 2539.755 | 255 | 720 | 1207 | 79 | 357 |
| 2543.193 | 229 | 714 | 1201 | 94 | 416 |
| 2546.63 | 265 | 709 | 1196 | 71 | 409 |
| 2550.065 | 249 | 706 | 1193 | 68 | 379 |
| 2553.499 | 261 | 754 | 1241 | 79 | 414 |
| 2556.931 | 283 | 758 | 1245 | 53 | 391 |
| 2560.362 | 245 | 779 | 1266 | 63 | 416 |
| 2563.792 | 248 | 767 | 1254 | 83 | 386 |
| 2567.219 | 264 | 782 | 1269 | 67 | 431 |
| 2570.646 | 283 | 783 | 1270 | 56 | 420 |
| 2574.071 | 255 | 820 | 1307 | 99 | 422 |
| 2577.494 | 290 | 756 | 1243 | 48 | 410 |
| 2580.916 | 250 | 882 | 1369 | 76 | 377 |
| 2584.337 | 255 | 897 | 1384 | 93 | 395 |
| 2587.756 | 284 | 787 | 1274 | 81 | 432 |
| 2591.173 | 253 | 879 | 1366 | 53 | 448 |
| 2594.589 | 263 | 929 | 1416 | 93 | 428 |
| 2598.004 | 250 | 942 | 1429 | 83 | 416 |
| 2601.417 | 257 | 893 | 1380 | 49 | 414 |
| 2604.829 | 251 | 1021 | 1508 | 78 | 403 |
| 2608.239 | 248 | 1048 | 1535 | 89 | 436 |
| 2611.648 | 250 | 995 | 1482 | 66 | 394 |
| 2615.055 | 271 | 1074 | 1561 | 63 | 392 |
| 2618.461 | 251 | 1154 | 1641 | 83 | 399 |
| 2621.865 | 272 | 1164 | 1651 | 79 | 431 |
| 2625.268 | 264 | 1165 | 1652 | 76 | 389 |
| 2628.67 | 276 | 1212 | 1727 | 78 | 401 |
| 2632.07 | 242 | 1240 | 1802 | 80 | 398 |
| 2635.468 | 244 | 1270 | 1876 | 46 | 425 |
| 2638.865 | 241 | 1294 | 1847 | 81 | 407 |
| 2642.261 | 253 | 1318 | 1960 | 78 | 397 |
| 2645.655 | 245 | 1337 | 2005 | 74 | 398 |
| 2649.048 | 238 | 1352 | 2192 | 70 | 386 |
| 2652.439 | 234 | 1372 | 2259 | 89 | 423 |
| 2655.829 | 230 | 1389 | 2295 | 52 | 430 |
| 2659.217 | 238 | 1398 | 2421 | 76 | 411 |
| 2662.604 | 231 | 1412 | 2567 | 70 | 411 |
| 2665.99 | 264 | 1422 | 2488 | 59 | 410 |
| 2669.373 | 266 | 1436 | 2666 | 79 | 411 |
| 2672.756 | 271 | 1448 | 2691 | 54 | 403 |
| 2676.137 | 256 | 1464 | 2780 | 54 | 424 |
| 2679.517 | 271 | 1485 | 2712 | 76 | 406 |
| 2682.895 | 254 | 1481 | 2784 | 66 | 415 |
| 2686.271 | 249 | 1476 | 2735 | 71 | 354 |
| 2689.647 | 266 | 1471 | 2738 | 64 | 427 |
| 2693.021 | 277 | 1465 | 2741 | 57 | 418 |
| 2696.393 | 251 | 1454 | 2641 | 46 | 379 |
| 2699.764 | 255 | 1446 | 2624 | 73 | 422 |
| 2703.133 | 253 | 1433 | 2492 | 51 | 412 |
| 2706.501 | 258 | 1420 | 2454 | 63 | 408 |
| 2709.868 | 261 | 1408 | 2315 | 67 | 370 |
| 2713.233 | 229 | 1380 | 2204 | 87 | 424 |
| 2716.597 | 234 | 1354 | 2128 | 55 | 409 |
| 2719.959 | 230 | 1335 | 2027 | 76 | 400 |
| 2723.32 | 267 | 1321 | 1964 | 52 | 445 |
| 2726.679 | 260 | 1309 | 1906 | 89 | 405 |
| 2730.038 | 229 | 1299 | 1860 | 65 | 413 |
| 2733.394 | 277 | 1282 | 1831 | 64 | 438 |
| 2736.749 | 244 | 1265 | 1800 | 67 | 363 |
| 2740.103 | 258 | 1245 | 1755 | 62 | 407 |
| 2743.455 | 265 | 1227 | 1714 | 59 | 417 |
| 2746.806 | 267 | 1193 | 1680 | 80 | 434 |
| 2750.156 | 274 | 1262 | 1749 | 68 | 429 |
| 2753.503 | 255 | 1133 | 1620 | 68 | 431 |
| 2756.85 | 251 | 1163 | 1650 | 61 | 413 |
| 2760.195 | 264 | 1107 | 1594 | 65 | 420 |
| 2763.539 | 222 | 1084 | 1571 | 97 | 397 |
| 2766.881 | 278 | 1136 | 1623 | 77 | 399 |
| 2770.222 | 239 | 1107 | 1594 | 81 | 410 |
| 2773.561 | 260 | 1094 | 1581 | 52 | 388 |
| 2776.899 | 208 | 989 | 1476 | 67 | 394 |
| 2780.236 | 259 | 1047 | 1534 | 56 | 459 |
| 2783.571 | 253 | 1046 | 1533 | 64 | 419 |
| 2786.905 | 250 | 1018 | 1505 | 78 | 400 |
| 2790.237 | 245 | 1044 | 1531 | 56 | 424 |
| 2793.568 | 244 | 1016 | 1503 | 66 | 448 |
| 2796.897 | 260 | 1101 | 1588 | 65 | 404 |
| 2800.226 | 249 | 1124 | 1611 | 78 | 398 |
| 2803.552 | 223 | 1105 | 1592 | 57 | 415 |
| 2806.877 | 268 | 1054 | 1541 | 71 | 380 |
| 2810.201 | 261 | 985 | 1472 | 79 | 404 |
| 2813.524 | 232 | 1060 | 1547 | 70 | 393 |
| 2816.844 | 275 | 1011 | 1498 | 57 | 369 |
| 2820.164 | 256 | 1103 | 1590 | 55 | 422 |
| 2823.482 | 251 | 1003 | 1490 | 47 | 401 |
| 2826.799 | 228 | 1041 | 1528 | 79 | 427 |
| 2830.115 | 255 | 1107 | 1594 | 84 | 425 |
| 2833.428 | 257 | 1070 | 1557 | 76 | 425 |
| 2836.741 | 237 | 1102 | 1589 | 65 | 430 |
| 2840.052 | 276 | 1064 | 1551 | 65 | 396 |
| 2843.362 | 268 | 1111 | 1598 | 67 | 449 |
| 2846.67 | 263 | 1141 | 1628 | 53 | 429 |
| 2849.977 | 261 | 1095 | 1582 | 61 | 423 |
| 2853.283 | 249 | 1173 | 1660 | 60 | 451 |
| 2856.587 | 263 | 1133 | 1620 | 71 | 408 |
| 2859.889 | 264 | 1152 | 1639 | 82 | 443 |
| 2863.191 | 280 | 1177 | 1664 | 58 | 419 |
| 2866.491 | 243 | 1172 | 1659 | 81 | 406 |
| 2869.789 | 255 | 1188 | 1675 | 54 | 453 |
| 2873.086 | 294 | 1202 | 1689 | 74 | 399 |
| 2876.382 | 273 | 1178 | 1665 | 76 | 421 |
| 2879.677 | 285 | 1239 | 1726 | 68 | 449 |
| 2882.969 | 273 | 1167 | 1654 | 72 | 411 |
| 2886.261 | 259 | 1237 | 1724 | 56 | 441 |
| 2889.551 | 245 | 1238 | 1725 | 69 | 428 |
| 2892.84 | 257 | 1188 | 1675 | 83 | 421 |
| 2896.127 | 257 | 1173 | 1660 | 54 | 398 |
| 2899.413 | 251 | 1282 | 1769 | 88 | 420 |
| 2902.698 | 292 | 1271 | 1758 | 65 | 419 |
| 2905.981 | 260 | 1200 | 1687 | 59 | 400 |
| 2909.263 | 263 | 1146 | 1633 | 52 | 410 |
| 2912.543 | 267 | 1224 | 1711 | 47 | 391 |
| 2915.822 | 259 | 1254 | 1741 | 68 | 449 |
| 2919.1 | 261 | 1233 | 1720 | 72 | 427 |
| 2922.376 | 262 | 1181 | 1668 | 70 | 421 |
| 2925.651 | 273 | 1177 | 1664 | 73 | 401 |
| 2928.925 | 263 | 1208 | 1695 | 83 | 428 |
| 2932.197 | 279 | 1249 | 1736 | 65 | 399 |
| 2935.468 | 277 | 1158 | 1645 | 67 | 448 |
| 2938.737 | 265 | 1195 | 1682 | 66 | 437 |
| 2942.005 | 271 | 1169 | 1656 | 72 | 435 |
| 2945.272 | 265 | 1129 | 1616 | 55 | 382 |
| 2948.537 | 261 | 1206 | 1693 | 63 | 434 |
| 2951.801 | 288 | 1080 | 1567 | 67 | 403 |
| 2955.063 | 274 | 1165 | 1652 | 92 | 403 |
| 2958.324 | 262 | 1034 | 1521 | 71 | 395 |
| 2961.584 | 265 | 996 | 1483 | 47 | 421 |
| 2964.843 | 258 | 1018 | 1505 | 66 | 400 |
| 2968.099 | 258 | 984 | 1471 | 69 | 431 |
| 2971.355 | 259 | 1008 | 1495 | 52 | 375 |
| 2974.609 | 251 | 880 | 1367 | 50 | 417 |
| 2977.862 | 260 | 908 | 1395 | 67 | 389 |
| 2981.114 | 249 | 899 | 1386 | 40 | 411 |
| 2984.364 | 278 | 928 | 1415 | 61 | 479 |
| 2987.613 | 266 | 846 | 1333 | 54 | 428 |
| 2990.86 | 267 | 895 | 1382 | 62 | 418 |
| 2994.106 | 266 | 877 | 1364 | 49 | 432 |
| 2997.351 | 238 | 768 | 1255 | 65 | 453 |
| 3000.594 | 284 | 832 | 1319 | 67 | 424 |
| 3003.836 | 251 | 839 | 1326 | 68 | 399 |
| 3007.077 | 259 | 850 | 1337 | 57 | 464 |
| 3010.316 | 238 | 842 | 1329 | 53 | 441 |
| 3013.554 | 236 | 773 | 1260 | 48 | 385 |
| 3016.79 | 240 | 772 | 1259 | 81 | 400 |
| 3020.025 | 286 | 757 | 1244 | 68 | 392 |
| 3023.259 | 250 | 682 | 1169 | 58 | 432 |
| 3026.492 | 284 | 696 | 1183 | 59 | 433 |
| 3029.723 | 267 | 721 | 1208 | 77 | 443 |
| 3032.953 | 249 | 679 | 1166 | 47 | 398 |
| 3036.181 | 257 | 699 | 1186 | 63 | 431 |
| 3039.408 | 248 | 667 | 1154 | 76 | 409 |
| 3042.634 | 262 | 692 | 1179 | 71 | 412 |
| 3045.858 | 255 | 656 | 1143 | 46 | 413 |
| 3049.081 | 259 | 668 | 1155 | 69 | 381 |
| 3052.302 | 264 | 758 | 1245 | 75 | 445 |
| 3055.523 | 241 | 610 | 1097 | 49 | 388 |
| 3058.742 | 277 | 559 | 1046 | 55 | 415 |
| 3061.959 | 263 | 577 | 1064 | 50 | 421 |
| 3065.176 | 235 | 731 | 1218 | 53 | 439 |
| 3068.39 | 248 | 597 | 1084 | 63 | 438 |
| 3071.604 | 250 | 608 | 1095 | 55 | 401 |
| 3074.816 | 258 | 640 | 1127 | 49 | 445 |
| 3078.027 | 259 | 670 | 1157 | 68 | 446 |
| 3081.237 | 251 | 570 | 1057 | 53 | 417 |
| 3084.445 | 264 | 566 | 1053 | 59 | 411 |
| 3087.652 | 268 | 574 | 1061 | 65 | 428 |
| 3090.857 | 295 | 616 | 1103 | 74 | 432 |
| 3094.061 | 270 | 626 | 1113 | 49 | 441 |
| 3097.264 | 264 | 577 | 1064 | 54 | 409 |
| 3100.466 | 254 | 692 | 1179 | 69 | 444 |
| 3103.666 | 266 | 573 | 1060 | 74 | 388 |
| 3106.865 | 247 | 599 | 1086 | 61 | 448 |
| 3110.062 | 259 | 616 | 1103 | 62 | 432 |
| 3113.258 | 248 | 553 | 1040 | 73 | 424 |
| 3116.453 | 254 | 586 | 1073 | 54 | 413 |
| 3119.646 | 281 | 541 | 1028 | 77 | 432 |
| 3122.838 | 234 | 578 | 1065 | 71 | 400 |
| 3126.029 | 277 | 607 | 1094 | 62 | 389 |
| 3129.219 | 235 | 576 | 1063 | 81 | 460 |
| 3132.407 | 283 | 589 | 1076 | 58 | 418 |
| 3135.594 | 272 | 624 | 1111 | 62 | 415 |
| 3138.779 | 277 | 569 | 1056 | 53 | 407 |
| 3141.963 | 248 | 623 | 1110 | 78 | 411 |
| 3145.146 | 258 | 513 | 1000 | 53 | 425 |
| 3148.328 | 236 | 576 | 1063 | 61 | 458 |
| 3151.508 | 281 | 578 | 1065 | 67 | 404 |
| 3154.687 | 257 | 559 | 1046 | 77 | 398 |
| 3157.864 | 263 | 576 | 1063 | 65 | 437 |
| 3161.041 | 256 | 546 | 1033 | 73 | 387 |
| 3164.215 | 254 | 583 | 1070 | 76 | 425 |
| 3167.389 | 259 | 529 | 1016 | 44 | 440 |
| 3170.561 | 301 | 564 | 1051 | 60 | 436 |
| 3173.732 | 238 | 573 | 1060 | 72 | 422 |
| 3176.902 | 268 | 556 | 1043 | 57 | 424 |
| 3180.07 | 269 | 522 | 1009 | 69 | 425 |
| 3183.237 | 271 | 511 | 998 | 70 | 440 |
| 3186.403 | 291 | 516 | 1003 | 69 | 414 |
| 3189.567 | 279 | 510 | 997 | 64 | 447 |
| 3192.73 | 234 | 496 | 983 | 71 | 436 |
| 3195.892 | 268 | 482 | 969 | 58 | 445 |
| 3199.052 | 253 | 534 | 1021 | 62 | 453 |
| 3202.211 | 274 | 503 | 990 | 41 | 436 |
| 3205.369 | 270 | 490 | 977 | 66 | 466 |
| 3208.526 | 238 | 486 | 973 | 59 | 476 |
| 3211.681 | 253 | 496 | 983 | 69 | 409 |
| 3214.834 | 281 | 448 | 935 | 77 | 430 |
| 3217.987 | 270 | 501 | 988 | 63 | 398 |
| 3221.138 | 240 | 483 | 970 | 39 | 443 |
| 3224.288 | 269 | 450 | 937 | 70 | 393 |
| 3227.437 | 266 | 461 | 948 | 70 | 430 |
| 3230.584 | 272 | 491 | 978 | 62 | 441 |
| 3233.73 | 232 | 492 | 979 | 67 | 473 |
| 3236.875 | 261 | 393 | 880 | 62 | 438 |
| 3240.018 | 283 | 438 | 925 | 49 | 451 |
| 3243.161 | 256 | 380 | 867 | 63 | 448 |
| 3246.301 | 240 | 389 | 876 | 69 | 431 |
| 3249.441 | 253 | 402 | 889 | 40 | 415 |
| 3252.579 | 304 | 326 | 813 | 56 | 447 |
| 3255.716 | 256 | 378 | 865 | 76 | 400 |
| 3258.852 | 263 | 357 | 844 | 65 | 407 |
| 3261.986 | 259 | 329 | 816 | 40 | 438 |
| 3265.119 | 278 | 265 | 752 | 68 | 428 |
| 3268.251 | 236 | 278 | 765 | 50 | 434 |
| 3271.381 | 263 | 315 | 802 | 75 | 423 |
| 3274.51 | 241 | 301 | 788 | 79 | 443 |
| 3277.638 | 247 | 272 | 759 | 82 | 424 |
| 3280.765 | 255 | 284 | 771 | 70 | 437 |
| 3283.89 | 261 | 296 | 783 | 65 | 420 |
| 3287.014 | 267 | 299 | 786 | 55 | 437 |
| 3290.137 | 249 | 272 | 759 | 59 | 405 |
| 3293.258 | 282 | 311 | 798 | 74 | 435 |
| 3296.378 | 245 | 287 | 774 | 62 | 432 |
| 3299.497 | 260 | 265 | 752 | 59 | 390 |
| 3302.615 | 286 | 267 | 754 | 59 | 438 |
| 3305.731 | 255 | 259 | 746 | 62 | 421 |
| 3308.846 | 274 | 294 | 781 | 61 | 469 |
| 3311.959 | 265 | 293 | 780 | 66 | 423 |
| 3315.072 | 243 | 255 | 742 | 56 | 391 |
| 3318.183 | 295 | 294 | 781 | 63 | 448 |
| 3321.293 | 258 | 327 | 814 | 60 | 456 |
| 3324.401 | 263 | 292 | 779 | 59 | 413 |
| 3327.509 | 277 | 296 | 783 | 58 | 423 |
| 3330.615 | 276 | 290 | 777 | 51 | 416 |
| 3333.719 | 257 | 298 | 785 | 74 | 417 |
| 3336.823 | 270 | 317 | 804 | 53 | 461 |
| 3339.925 | 298 | 262 | 749 | 73 | 375 |
| 3343.026 | 270 | 228 | 715 | 70 | 458 |
| 3346.125 | 245 | 285 | 772 | 63 | 440 |
| 3349.224 | 240 | 251 | 738 | 57 | 432 |
| 3352.321 | 282 | 280 | 767 | 62 | 419 |
| 3355.417 | 241 | 289 | 776 | 56 | 445 |
| 3358.511 | 286 | 264 | 751 | 70 | 418 |
| 3361.604 | 263 | 268 | 755 | 73 | 419 |
| 3364.697 | 285 | 224 | 711 | 55 | 424 |
| 3367.787 | 230 | 275 | 762 | 66 | 409 |
| 3370.877 | 273 | 275 | 762 | 68 | 399 |
| 3373.965 | 240 | 274 | 761 | 62 | 454 |
| 3377.052 | 241 | 259 | 746 | 74 | 450 |
| 3380.137 | 254 | 333 | 820 | 56 | 422 |
| 3383.222 | 235 | 247 | 734 | 45 | 422 |
| 3386.305 | 267 | 263 | 750 | 67 | 438 |
| 3389.387 | 269 | 275 | 762 | 58 | 427 |
| 3392.467 | 235 | 266 | 753 | 65 | 412 |
| 3395.547 | 249 | 261 | 748 | 49 | 429 |
| 3398.625 | 269 | 240 | 727 | 68 | 452 |
| 3401.701 | 226 | 299 | 786 | 59 | 440 |
| 3404.777 | 262 | 302 | 789 | 55 | 428 |
| 3407.851 | 247 | 282 | 769 | 46 | 420 |
| 3410.924 | 309 | 284 | 771 | 50 | 461 |
| 3413.996 | 266 | 218 | 705 | 51 | 427 |
| 3417.067 | 237 | 260 | 747 | 77 | 459 |
| 3420.136 | 244 | 247 | 734 | 46 | 463 |
| 3423.204 | 245 | 279 | 766 | 45 | 406 |
| 3426.271 | 267 | 259 | 746 | 68 | 441 |
| 3429.336 | 253 | 251 | 738 | 56 | 459 |
| 3432.4 | 282 | 212 | 699 | 80 | 407 |
| 3435.463 | 244 | 280 | 767 | 58 | 436 |
| 3438.525 | 252 | 295 | 782 | 60 | 431 |
| 3441.585 | 276 | 285 | 772 | 53 | 457 |
| 3444.645 | 248 | 309 | 796 | 74 | 461 |
| 3447.702 | 232 | 227 | 714 | 77 | 425 |
| 3450.759 | 265 | 223 | 710 | 68 | 399 |
| 3453.815 | 249 | 259 | 746 | 53 | 476 |
| 3456.869 | 255 | 256 | 743 | 59 | 441 |
| 3459.922 | 252 | 277 | 764 | 64 | 382 |
| 3462.973 | 279 | 259 | 746 | 65 | 431 |
| 3466.024 | 265 | 252 | 739 | 70 | 418 |
| 3469.073 | 245 | 260 | 747 | 59 | 447 |
| 3472.121 | 243 | 235 | 722 | 58 | 397 |
| 3475.168 | 241 | 210 | 697 | 56 | 402 |
| 3478.213 | 253 | 263 | 750 | 58 | 438 |
| 3481.258 | 282 | 205 | 692 | 58 | 406 |
| 3484.301 | 244 | 246 | 733 | 57 | 403 |
| 3487.343 | 258 | 199 | 686 | 52 | 411 |
| 3490.383 | 274 | 268 | 755 | 43 | 448 |
| 3493.422 | 267 | 223 | 710 | 54 | 426 |
| 3496.46 | 272 | 228 | 715 | 77 | 450 |
| 3499.497 | 237 | 259 | 746 | 52 | 399 |
| 3502.533 | 260 | 206 | 693 | 51 | 435 |
| 3505.567 | 227 | 212 | 699 | 55 | 426 |
| 3508.6 | 252 | 276 | 763 | 49 | 429 |
| 3511.632 | 254 | 257 | 744 | 70 | 378 |
| 3514.663 | 238 | 303 | 790 | 63 | 407 |
| 3517.692 | 251 | 236 | 723 | 51 | 471 |
| 3520.72 | 242 | 192 | 679 | 70 | 410 |
| 3523.747 | 262 | 219 | 706 | 63 | 454 |
| 3526.773 | 243 | 274 | 761 | 49 | 434 |
| 3529.797 | 259 | 248 | 735 | 61 | 387 |
| 3532.821 | 274 | 277 | 764 | 64 | 424 |
| 3535.843 | 225 | 211 | 698 | 60 | 435 |
| 3538.864 | 254 | 220 | 707 | 59 | 450 |
| 3541.883 | 242 | 205 | 692 | 46 | 431 |
| 3544.901 | 267 | 264 | 751 | 54 | 427 |
| 3547.918 | 223 | 249 | 736 | 66 | 427 |
| 3550.934 | 279 | 276 | 763 | 54 | 395 |
| 3553.949 | 255 | 269 | 756 | 54 | 417 |
| 3556.962 | 258 | 256 | 743 | 45 | 445 |
| 3559.975 | 248 | 279 | 766 | 62 | 404 |
| 3562.986 | 265 | 254 | 741 | 63 | 392 |
| 3565.995 | 256 | 238 | 725 | 55 | 432 |
| 3569.004 | 239 | 223 | 710 | 63 | 443 |
| 3572.011 | 233 | 245 | 732 | 60 | 442 |
| 3575.017 | 241 | 218 | 705 | 66 | 484 |
| 3578.022 | 259 | 262 | 749 | 61 | 446 |
| 3581.026 | 261 | 250 | 737 | 55 | 414 |
| 3584.028 | 263 | 227 | 714 | 61 | 451 |
| 3587.03 | 267 | 232 | 719 | 49 | 450 |
| 3590.03 | 205 | 250 | 737 | 56 | 400 |
| 3593.029 | 250 | 264 | 751 | 66 | 425 |
| 3596.026 | 251 | 249 | 736 | 73 | 411 |
| 3599.022 | 243 | 262 | 749 | 64 | 409 |
| 3602.018 | 238 | 219 | 706 | 56 | 442 |
| 3605.012 | 259 | 295 | 782 | 73 | 437 |
| 3608.004 | 264 | 198 | 685 | 54 | 434 |
| 3610.996 | 240 | 267 | 754 | 66 | 458 |
| 3613.986 | 225 | 236 | 723 | 60 | 449 |
| 3616.975 | 255 | 244 | 731 | 69 | 424 |
| 3619.963 | 248 | 194 | 681 | 67 | 405 |
| 3622.95 | 232 | 241 | 728 | 62 | 465 |
| 3625.936 | 249 | 259 | 746 | 50 | 394 |
| 3628.92 | 249 | 220 | 707 | 62 | 438 |
| 3631.903 | 270 | 220 | 707 | 62 | 427 |
| 3634.885 | 230 | 234 | 721 | 62 | 429 |
| 3637.865 | 286 | 231 | 718 | 50 | 394 |
| 3640.845 | 236 | 271 | 758 | 59 | 446 |
| 3643.823 | 232 | 283 | 770 | 71 | 420 |
| 3646.8 | 222 | 226 | 713 | 55 | 453 |
| 3649.776 | 250 | 218 | 705 | 65 | 437 |
| 3652.751 | 243 | 270 | 757 | 64 | 426 |
| 3655.724 | 256 | 206 | 693 | 71 | 424 |
| 3658.696 | 271 | 245 | 732 | 72 | 431 |
| 3661.667 | 246 | 270 | 757 | 51 | 446 |
| 3664.637 | 275 | 269 | 756 | 45 | 446 |
| 3667.606 | 216 | 265 | 752 | 52 | 415 |
| 3670.573 | 272 | 284 | 771 | 57 | 407 |
| 3673.54 | 248 | 240 | 727 | 55 | 428 |
| 3676.505 | 248 | 282 | 769 | 71 | 406 |
| 3679.469 | 239 | 324 | 811 | 63 | 406 |
| 3682.431 | 254 | 227 | 714 | 51 | 410 |
| 3685.393 | 264 | 346 | 833 | 63 | 409 |
| 3688.353 | 251 | 298 | 785 | 55 | 416 |
| 3691.312 | 222 | 241 | 728 | 56 | 399 |
| 3694.27 | 247 | 259 | 746 | 66 | 412 |
| 3697.227 | 207 | 264 | 751 | 59 | 402 |
| 3700.183 | 229 | 322 | 809 | 50 | 441 |
| 3703.137 | 227 | 306 | 793 | 41 | 391 |
| 3706.09 | 232 | 302 | 789 | 67 | 418 |
| 3709.042 | 245 | 241 | 728 | 58 | 462 |
| 3711.993 | 264 | 207 | 694 | 60 | 435 |
| 3714.943 | 236 | 265 | 752 | 52 | 409 |
| 3717.891 | 214 | 313 | 800 | 65 | 458 |
| 3720.838 | 213 | 279 | 766 | 49 | 442 |
| 3723.784 | 225 | 280 | 767 | 64 | 412 |
| 3726.729 | 248 | 242 | 729 | 40 | 466 |
| 3729.673 | 260 | 212 | 699 | 51 | 411 |
| 3732.615 | 236 | 182 | 669 | 64 | 386 |
| 3735.557 | 252 | 275 | 762 | 51 | 453 |
| 3738.497 | 239 | 304 | 791 | 66 | 440 |
| 3741.436 | 231 | 270 | 757 | 62 | 427 |
| 3744.374 | 241 | 256 | 743 | 65 | 393 |
| 3747.31 | 222 | 191 | 678 | 53 | 416 |
| 3750.246 | 225 | 239 | 726 | 55 | 399 |
| 3753.18 | 237 | 230 | 717 | 48 | 389 |
| 3756.113 | 266 | 301 | 788 | 78 | 398 |
| 3759.045 | 214 | 267 | 754 | 64 | 462 |
| 3761.976 | 246 | 246 | 733 | 51 | 408 |
| 3764.905 | 233 | 259 | 746 | 65 | 422 |
| 3767.833 | 262 | 293 | 780 | 56 | 421 |
| 3770.761 | 220 | 274 | 761 | 59 | 414 |
| 3773.687 | 222 | 308 | 795 | 55 | 388 |
| 3776.612 | 227 | 242 | 729 | 48 | 437 |
| 3779.535 | 236 | 257 | 744 | 54 | 439 |
| 3782.458 | 264 | 262 | 749 | 56 | 402 |
| 3785.379 | 222 | 268 | 755 | 61 | 421 |
| 3788.3 | 212 | 254 | 741 | 64 | 441 |
| 3791.219 | 225 | 259 | 746 | 71 | 440 |
| 3794.136 | 241 | 250 | 737 | 48 | 415 |
| 3797.053 | 232 | 273 | 760 | 47 | 422 |
| 3799.969 | 235 | 258 | 745 | 75 | 444 |
| 3802.883 | 223 | 265 | 752 | 49 | 420 |
| 3805.796 | 229 | 234 | 721 | 54 | 388 |
| 3808.708 | 239 | 287 | 774 | 60 | 414 |
| 3811.619 | 208 | 286 | 773 | 62 | 456 |
| 3814.529 | 203 | 270 | 757 | 52 | 431 |
| 3817.437 | 250 | 264 | 751 | 60 | 447 |
| 3820.345 | 221 | 217 | 704 | 59 | 443 |
| 3823.251 | 231 | 270 | 757 | 67 | 419 |
| 3826.156 | 235 | 277 | 764 | 53 | 392 |
| 3829.06 | 209 | 247 | 734 | 53 | 383 |
| 3831.963 | 237 | 282 | 769 | 42 | 440 |
| 3834.864 | 194 | 209 | 696 | 71 | 445 |
| 3837.765 | 223 | 267 | 754 | 45 | 394 |
| 3840.664 | 201 | 267 | 754 | 49 | 442 |
| 3843.562 | 218 | 204 | 691 | 47 | 435 |
| 3846.459 | 225 | 271 | 758 | 46 | 401 |
| 3849.355 | 225 | 328 | 815 | 57 | 406 |
| 3852.25 | 241 | 272 | 759 | 63 | 500 |
| 3855.143 | 224 | 269 | 756 | 58 | 390 |
| 3858.035 | 222 | 254 | 741 | 58 | 404 |
| 3860.927 | 235 | 295 | 782 | 51 | 410 |
| 3863.817 | 228 | 289 | 776 | 55 | 383 |
| 3866.706 | 214 | 267 | 754 | 59 | 436 |
| 3869.593 | 231 | 216 | 703 | 52 | 416 |
| 3872.48 | 219 | 279 | 766 | 45 | 415 |
| 3875.365 | 241 | 245 | 732 | 64 | 417 |
| 3878.25 | 204 | 236 | 723 | 71 | 377 |
| 3881.133 | 217 | 253 | 740 | 52 | 442 |
| 3884.015 | 211 | 230 | 717 | 55 | 403 |
| 3886.896 | 199 | 263 | 750 | 49 | 401 |
| 3889.776 | 258 | 255 | 742 | 52 | 431 |
| 3892.654 | 212 | 294 | 781 | 51 | 409 |
| 3895.531 | 228 | 272 | 759 | 61 | 359 |
| 3898.408 | 190 | 274 | 761 | 51 | 409 |
| 3901.283 | 218 | 259 | 746 | 54 | 435 |
| 3904.157 | 254 | 328 | 815 | 55 | 434 |
| 3907.03 | 215 | 306 | 793 | 78 | 433 |
| 3909.902 | 221 | 299 | 786 | 39 | 395 |
| 3912.772 | 247 | 260 | 747 | 74 | 421 |
| 3915.642 | 218 | 245 | 732 | 52 | 402 |
| 3918.51 | 204 | 287 | 774 | 55 | 393 |
| 3921.377 | 211 | 259 | 746 | 63 | 430 |
| 3924.243 | 234 | 303 | 790 | 58 | 401 |
| 3927.108 | 239 | 318 | 805 | 64 | 460 |
| 3929.972 | 223 | 348 | 835 | 54 | 418 |
| 3932.834 | 233 | 260 | 747 | 64 | 400 |
| 3935.696 | 200 | 310 | 797 | 47 | 443 |
| 3938.556 | 210 | 319 | 806 | 61 | 374 |
| 3941.416 | 251 | 334 | 821 | 57 | 414 |
| 3944.274 | 201 | 273 | 760 | 51 | 384 |
| 3947.131 | 218 | 301 | 788 | 42 | 455 |
| 3949.987 | 235 | 234 | 721 | 38 | 433 |
| 3952.841 | 253 | 299 | 786 | 53 | 445 |
| 3955.695 | 248 | 310 | 797 | 48 | 428 |
| 3958.547 | 222 | 261 | 748 | 58 | 402 |
| 3961.399 | 228 | 324 | 811 | 62 | 424 |
| 3964.249 | 230 | 277 | 764 | 47 | 433 |
| 3967.098 | 202 | 319 | 806 | 51 | 414 |
| 3969.946 | 230 | 295 | 782 | 41 | 412 |
| 3972.793 | 234 | 255 | 742 | 62 | 427 |
| 3975.638 | 207 | 259 | 746 | 45 | 417 |
| 3978.483 | 192 | 303 | 790 | 57 | 402 |
| 3981.326 | 205 | 341 | 828 | 67 | 419 |
| 3984.169 | 218 | 318 | 805 | 52 | 426 |
| 3987.01 | 214 | 321 | 808 | 52 | 390 |
| 3989.85 | 224 | 317 | 804 | 55 | 429 |
| 3992.689 | 216 | 291 | 778 | 58 | 390 |
| 3995.527 | 200 | 293 | 780 | 58 | 415 |
| 3998.364 | 230 | 362 | 849 | 55 | 450 |
| 4001.199 | 226 | 269 | 756 | 51 | 408 |
| 4004.034 | 238 | 352 | 839 | 65 | 397 |
| 4006.867 | 203 | 275 | 762 | 66 | 384 |
| 4009.699 | 215 | 337 | 824 | 69 | 408 |
| 4012.53 | 236 | 303 | 790 | 56 | 426 |
| 4015.36 | 205 | 284 | 771 | 70 | 370 |
| 4018.189 | 210 | 339 | 826 | 65 | 402 |
| 4021.017 | 227 | 343 | 830 | 65 | 421 |
| 4023.844 | 212 | 287 | 774 | 42 | 405 |
| 4026.669 | 202 | 253 | 740 | 58 | 380 |
| 4029.494 | 185 | 307 | 794 | 42 | 419 |
| 4032.317 | 190 | 347 | 834 | 53 | 368 |
| 4035.139 | 199 | 256 | 743 | 59 | 428 |
| 4037.96 | 226 | 313 | 800 | 61 | 409 |
| 4040.781 | 208 | 359 | 846 | 44 | 403 |
| 4043.599 | 190 | 280 | 767 | 53 | 411 |
| 4046.417 | 187 | 305 | 792 | 70 | 410 |
| 4049.234 | 214 | 338 | 825 | 60 | 385 |
| 4052.049 | 221 | 377 | 864 | 54 | 395 |
| 4054.864 | 226 | 332 | 819 | 56 | 381 |
| 4057.677 | 209 | 298 | 785 | 49 | 406 |
| 4060.489 | 209 | 288 | 775 | 47 | 411 |
| 4063.301 | 191 | 317 | 804 | 57 | 392 |
| 4066.111 | 203 | 267 | 754 | 60 | 379 |
| 4068.919 | 228 | 306 | 793 | 52 | 405 |
| 4071.727 | 176 | 315 | 802 | 58 | 405 |
| 4074.534 | 221 | 254 | 741 | 53 | 365 |
| 4077.34 | 191 | 318 | 805 | 65 | 414 |
| 4080.144 | 198 | 292 | 779 | 49 | 417 |
| 4082.948 | 221 | 287 | 774 | 58 | 428 |
| 4085.75 | 217 | 387 | 874 | 55 | 395 |
| 4088.551 | 227 | 267 | 754 | 60 | 412 |
| 4091.351 | 227 | 319 | 806 | 55 | 386 |
| 4094.15 | 225 | 360 | 847 | 43 | 372 |
| 4096.948 | 211 | 330 | 817 | 75 | 381 |
| 4099.745 | 195 | 286 | 773 | 48 | 424 |
| 4102.541 | 205 | 345 | 832 | 42 | 370 |
| 4105.335 | 195 | 285 | 772 | 58 | 394 |
| 4108.129 | 206 | 298 | 785 | 49 | 406 |
| 4110.921 | 217 | 303 | 790 | 36 | 400 |
| 4113.713 | 209 | 323 | 810 | 46 | 363 |
| 4116.503 | 212 | 338 | 825 | 56 | 422 |
| 4119.292 | 217 | 316 | 803 | 52 | 423 |
| 4122.081 | 222 | 298 | 785 | 46 | 387 |
| 4124.867 | 229 | 298 | 785 | 45 | 402 |
| 4127.653 | 197 | 365 | 852 | 54 | 448 |
| 4130.438 | 209 | 330 | 817 | 54 | 390 |
| 4133.222 | 209 | 313 | 800 | 58 | 440 |
| 4136.004 | 190 | 330 | 817 | 75 | 412 |
| 4138.786 | 184 | 297 | 784 | 48 | 423 |
| 4141.566 | 207 | 290 | 777 | 60 | 383 |
| 4144.346 | 196 | 376 | 863 | 36 | 400 |
| 4147.124 | 213 | 354 | 841 | 48 | 411 |
| 4149.901 | 216 | 347 | 834 | 64 | 396 |
| 4152.678 | 223 | 364 | 851 | 53 | 376 |
| 4155.453 | 171 | 372 | 859 | 50 | 375 |
| 4158.227 | 204 | 343 | 830 | 69 | 436 |
| 4161 | 190 | 331 | 818 | 44 | 403 |
| 4163.771 | 185 | 366 | 853 | 49 | 384 |
| 4166.542 | 176 | 316 | 803 | 59 | 400 |
| 4169.312 | 199 | 328 | 815 | 58 | 403 |
| 4172.08 | 194 | 351 | 838 | 49 | 383 |
| 4174.848 | 213 | 340 | 827 | 54 | 407 |
| 4177.614 | 197 | 389 | 876 | 48 | 378 |
| 4180.379 | 208 | 315 | 802 | 54 | 403 |
| 4183.144 | 207 | 366 | 853 | 59 | 412 |
| 4185.907 | 209 | 342 | 829 | 46 | 390 |
| 4188.668 | 188 | 292 | 779 | 60 | 401 |
| 4191.43 | 214 | 325 | 812 | 56 | 403 |
| 4194.189 | 196 | 384 | 871 | 56 | 406 |
| 4196.948 | 169 | 341 | 828 | 60 | 424 |
| 4199.706 | 195 | 366 | 853 | 55 | 361 |
| 4202.463 | 193 | 389 | 876 | 51 | 371 |
| 4205.218 | 194 | 368 | 855 | 41 | 364 |
| 4207.973 | 210 | 424 | 911 | 58 | 442 |
| 4210.727 | 196 | 429 | 916 | 54 | 409 |
| 4213.479 | 181 | 422 | 909 | 50 | 393 |
| 4216.23 | 222 | 409 | 896 | 50 | 417 |
| 4218.98 | 200 | 477 | 964 | 73 | 386 |
| 4221.73 | 214 | 457 | 944 | 46 | 425 |
| 4224.478 | 171 | 430 | 917 | 75 | 414 |
| 4227.225 | 192 | 439 | 926 | 51 | 381 |
| 4229.971 | 193 | 451 | 938 | 46 | 356 |
| 4232.716 | 203 | 419 | 906 | 61 | 416 |
| 4235.46 | 222 | 471 | 958 | 49 | 391 |
| 4238.203 | 214 | 411 | 898 | 51 | 409 |
| 4240.944 | 191 | 422 | 909 | 51 | 381 |
| 4243.685 | 191 | 416 | 903 | 52 | 389 |
| 4246.424 | 192 | 442 | 929 | 54 | 365 |
| 4249.163 | 208 | 455 | 942 | 53 | 402 |
| 4251.9 | 181 | 402 | 889 | 50 | 417 |
| 4254.637 | 211 | 438 | 925 | 61 | 392 |
| 4257.373 | 179 | 455 | 942 | 64 | 353 |
| 4260.106 | 201 | 443 | 930 | 49 | 388 |
| 4262.84 | 197 | 446 | 933 | 65 | 423 |
| 4265.572 | 176 | 420 | 907 | 52 | 391 |
| 4268.303 | 212 | 454 | 941 | 73 | 401 |
| 4271.033 | 190 | 484 | 971 | 49 | 396 |
| 4273.762 | 196 | 479 | 966 | 57 | 411 |
| 4276.49 | 196 | 456 | 943 | 56 | 383 |
| 4279.217 | 186 | 498 | 985 | 62 | 411 |
| 4281.943 | 197 | 494 | 981 | 54 | 395 |
| 4284.667 | 192 | 415 | 902 | 48 | 400 |
| 4287.391 | 208 | 441 | 928 | 52 | 427 |
| 4290.114 | 242 | 397 | 884 | 65 | 368 |
| 4292.835 | 197 | 397 | 884 | 64 | 387 |
| 4295.556 | 207 | 445 | 932 | 60 | 388 |
| 4298.276 | 203 | 491 | 978 | 65 | 388 |
| 4300.994 | 190 | 457 | 944 | 66 | 350 |
| 4303.711 | 216 | 388 | 875 | 62 | 373 |
| 4306.428 | 188 | 443 | 930 | 76 | 404 |
| 4309.143 | 189 | 415 | 902 | 72 | 378 |
| 4311.857 | 166 | 382 | 869 | 68 | 336 |
| 4314.571 | 153 | 434 | 921 | 64 | 386 |
| 4317.283 | 181 | 373 | 860 | 61 | 391 |
| 4319.994 | 198 | 413 | 900 | 53 | 384 |
| 4322.704 | 215 | 364 | 851 | 66 | 356 |
| 4325.413 | 186 | 337 | 824 | 54 | 378 |
| 4328.121 | 217 | 356 | 843 | 68 | 425 |
| 4330.828 | 185 | 302 | 789 | 57 | 406 |
| 4333.534 | 207 | 309 | 796 | 75 | 386 |
| 4336.239 | 192 | 330 | 817 | 54 | 362 |
| 4338.942 | 210 | 373 | 860 | 48 | 402 |
| 4341.645 | 185 | 342 | 829 | 59 | 370 |
| 4344.347 | 174 | 335 | 822 | 56 | 360 |
| 4347.047 | 166 | 340 | 827 | 45 | 374 |
| 4349.747 | 203 | 286 | 773 | 65 | 373 |
| 4352.446 | 179 | 326 | 813 | 68 | 377 |
| 4355.143 | 180 | 322 | 809 | 54 | 378 |
| 4357.84 | 183 | 316 | 803 | 68 | 387 |
| 4360.535 | 193 | 289 | 776 | 58 | 368 |
| 4363.229 | 200 | 330 | 817 | 44 | 365 |
| 4365.923 | 194 | 337 | 824 | 61 | 384 |
| 4368.615 | 196 | 279 | 766 | 70 | 376 |
| 4371.307 | 176 | 255 | 742 | 52 | 367 |
| 4373.997 | 189 | 311 | 798 | 52 | 400 |
| 4376.686 | 164 | 342 | 829 | 62 | 380 |
| 4379.375 | 182 | 341 | 828 | 47 | 413 |
| 4382.062 | 179 | 319 | 806 | 61 | 390 |
| 4384.748 | 176 | 320 | 807 | 43 | 393 |
| 4387.433 | 175 | 312 | 799 | 41 | 392 |
| 4390.117 | 193 | 299 | 786 | 59 | 390 |
| 4392.8 | 187 | 297 | 784 | 47 | 418 |
| 4395.482 | 195 | 270 | 757 | 65 | 390 |
| 4398.163 | 158 | 308 | 795 | 57 | 369 |
| 4400.843 | 211 | 269 | 756 | 61 | 400 |
| 4403.522 | 202 | 315 | 802 | 36 | 382 |
| 4406.2 | 209 | 219 | 706 | 56 | 364 |
| 4408.876 | 173 | 306 | 793 | 36 | 386 |
| 4411.552 | 164 | 342 | 829 | 42 | 431 |
| 4414.227 | 179 | 284 | 771 | 69 | 417 |
| 4416.901 | 181 | 283 | 770 | 48 | 379 |
| 4419.574 | 168 | 297 | 784 | 41 | 358 |
| 4422.246 | 184 | 349 | 836 | 54 | 403 |
| 4424.916 | 196 | 264 | 751 | 48 | 350 |
| 4427.586 | 182 | 278 | 765 | 37 | 394 |
| 4430.254 | 176 | 252 | 739 | 49 | 373 |
| 4432.922 | 183 | 231 | 718 | 42 | 380 |
| 4435.589 | 190 | 230 | 717 | 47 | 367 |
| 4438.254 | 173 | 290 | 777 | 40 | 345 |
| 4440.919 | 190 | 308 | 795 | 54 | 388 |
| 4443.583 | 161 | 264 | 751 | 57 | 381 |
| 4446.245 | 172 | 244 | 731 | 36 | 377 |
| 4448.906 | 164 | 277 | 764 | 33 | 409 |
| 4451.567 | 199 | 267 | 754 | 46 | 392 |
| 4454.227 | 171 | 253 | 740 | 56 | 349 |
| 4456.885 | 191 | 236 | 723 | 48 | 372 |
| 4459.542 | 198 | 281 | 768 | 49 | 388 |
| 4462.199 | 160 | 264 | 751 | 47 | 402 |
| 4464.854 | 183 | 265 | 752 | 35 | 379 |
| 4467.508 | 194 | 260 | 747 | 41 | 369 |
| 4470.162 | 181 | 274 | 761 | 37 | 349 |
| 4472.814 | 156 | 242 | 729 | 45 | 376 |
| 4475.465 | 174 | 255 | 742 | 33 | 371 |
| 4478.116 | 163 | 213 | 700 | 41 | 394 |
| 4480.765 | 190 | 274 | 761 | 46 | 360 |
| 4483.414 | 181 | 300 | 787 | 25 | 374 |
| 4486.061 | 163 | 291 | 778 | 52 | 371 |
| 4488.707 | 159 | 282 | 769 | 49 | 350 |
| 4491.353 | 164 | 224 | 711 | 36 | 359 |
| 4493.997 | 170 | 243 | 730 | 49 | 373 |
| 4496.64 | 171 | 246 | 733 | 43 | 372 |
| 4499.282 | 172 | 261 | 748 | 43 | 384 |
| 4501.923 | 167 | 239 | 726 | 50 | 351 |
| 4504.563 | 171 | 247 | 734 | 42 | 347 |
| 4507.203 | 178 | 199 | 686 | 58 | 378 |
| 4509.841 | 169 | 255 | 742 | 46 | 332 |
| 4512.478 | 177 | 243 | 730 | 53 | 355 |
| 4515.114 | 155 | 239 | 726 | 51 | 408 |
| 4517.75 | 181 | 235 | 722 | 56 | 373 |
| 4520.384 | 161 | 263 | 750 | 50 | 383 |
| 4523.017 | 186 | 254 | 741 | 44 | 350 |
| 4525.649 | 167 | 258 | 745 | 45 | 357 |
| 4528.28 | 155 | 266 | 753 | 42 | 353 |
| 4530.911 | 209 | 226 | 713 | 43 | 379 |
| 4533.54 | 148 | 219 | 706 | 59 | 345 |
| 4536.168 | 172 | 215 | 702 | 45 | 354 |
| 4538.795 | 179 | 238 | 725 | 37 | 388 |
| 4541.421 | 157 | 261 | 748 | 28 | 368 |
| 4544.046 | 157 | 225 | 712 | 43 | 362 |
| 4546.67 | 172 | 229 | 716 | 33 | 358 |
| 4549.293 | 161 | 201 | 688 | 46 | 368 |
| 4551.916 | 173 | 198 | 685 | 44 | 373 |
| 4554.537 | 176 | 237 | 724 | 48 | 337 |
| 4557.157 | 182 | 225 | 712 | 60 | 367 |
| 4559.776 | 165 | 239 | 726 | 34 | 388 |
| 4562.395 | 137 | 176 | 663 | 50 | 336 |
| 4565.012 | 153 | 241 | 728 | 41 | 347 |
| 4567.628 | 150 | 211 | 698 | 37 | 328 |
| 4570.243 | 159 | 225 | 712 | 60 | 363 |
| 4572.857 | 172 | 208 | 695 | 53 | 395 |
| 4575.471 | 156 | 164 | 651 | 45 | 369 |
| 4578.083 | 161 | 170 | 657 | 40 | 348 |
| 4580.694 | 157 | 238 | 725 | 42 | 337 |
| 4583.304 | 153 | 237 | 724 | 52 | 351 |
| 4585.914 | 153 | 173 | 660 | 49 | 330 |
| 4588.522 | 164 | 181 | 668 | 21 | 369 |
| 4591.129 | 168 | 213 | 700 | 52 | 357 |
| 4593.735 | 157 | 191 | 678 | 36 | 324 |
| 4596.341 | 154 | 200 | 687 | 47 | 395 |
| 4598.945 | 170 | 201 | 688 | 62 | 339 |
| 4601.548 | 162 | 248 | 735 | 34 | 354 |
| 4604.151 | 185 | 272 | 759 | 52 | 351 |
| 4606.752 | 148 | 162 | 649 | 50 | 387 |
| 4609.353 | 161 | 228 | 715 | 28 | 386 |
| 4611.952 | 163 | 148 | 635 | 43 | 350 |
| 4614.55 | 167 | 155 | 642 | 49 | 324 |
| 4617.148 | 161 | 197 | 684 | 45 | 337 |
| 4619.744 | 161 | 189 | 676 | 46 | 341 |
| 4622.34 | 174 | 178 | 665 | 50 | 365 |
| 4624.934 | 154 | 153 | 640 | 51 | 353 |
| 4627.527 | 153 | 174 | 661 | 48 | 344 |
| 4630.12 | 153 | 157 | 644 | 30 | 369 |
| 4632.711 | 154 | 198 | 685 | 46 | 311 |
| 4635.302 | 161 | 146 | 633 | 51 | 361 |
| 4637.892 | 186 | 181 | 668 | 62 | 340 |
| 4640.48 | 194 | 182 | 669 | 38 | 376 |
| 4643.068 | 166 | 159 | 646 | 42 | 354 |
| 4645.655 | 141 | 203 | 690 | 54 | 334 |
| 4648.24 | 156 | 195 | 682 | 44 | 375 |
| 4650.825 | 146 | 184 | 671 | 55 | 341 |
| 4653.409 | 155 | 189 | 676 | 51 | 377 |
| 4655.991 | 177 | 154 | 641 | 37 | 349 |
| 4658.573 | 172 | 215 | 702 | 39 | 344 |
| 4661.154 | 166 | 190 | 677 | 48 | 352 |
| 4663.733 | 174 | 163 | 650 | 31 | 325 |
| 4666.313 | 133 | 192 | 679 | 33 | 328 |
| 4668.89 | 154 | 202 | 689 | 51 | 335 |
| 4671.467 | 144 | 186 | 673 | 45 | 335 |
| 4674.043 | 153 | 168 | 655 | 52 | 340 |
| 4676.618 | 148 | 117 | 604 | 42 | 328 |
| 4679.192 | 151 | 186 | 673 | 40 | 365 |
| 4681.765 | 169 | 208 | 695 | 67 | 353 |
| 4684.337 | 169 | 160 | 647 | 46 | 348 |
| 4686.908 | 181 | 185 | 672 | 47 | 353 |
| 4689.478 | 145 | 196 | 683 | 46 | 321 |
| 4692.047 | 149 | 205 | 692 | 35 | 333 |
